# Integrative Proteomic and Lipidomic Analysis of Patients With Acute Myocardial Infarction Treated With PCSK9 Antibodies and Statins

**DOI:** 10.1161/CIRCGEN.125.005345

**Published:** 2026-02-10

**Authors:** Lukas E. Schmidt, Sean A. Burnap, Bhawana Singh, Kaloyan Takov, Sylvain Losdat, Lore Schrutka, Lukas Galli, Konstantinos Theofilatos, Georg W. Otto, Christian Hengstenberg, Ioanna Tzoulaki, Irene M. Lang, Konstantinos C. Koskinas, Walter S. Speidl, Lorenz Räber, Manuel Mayr

**Affiliations:** 1 Division of Cardiology, Department of Internal Medicine II, Medical University of Vienna, Austria (L.E.S., L.S., L.G., C.H., I.M.L., W.S.S., M.M.).; 2 Department of Biochemistry, University of Oxford, United Kingdom (S.A.B.).; 3 The Kavli Institute for Nanoscience Discovery, University of Oxford, United Kingdom (S.A.B.).; 4 National Heart and Lung Institute (B.S., K. Takov, M.M.), Imperial College London, United Kingdom.; 5 Department of Epidemiology and Biostatistics, School of Public Health (G.W.O., I.T.), Imperial College London, United Kingdom.; 6 Department of Clinical Research (S.L.), University of Bern, Switzerland.; 7 Department of Cardiology (K.C.K., L.R.), University of Bern, Switzerland.; 8 British Heart Foundation Centre of Research Excellence, School of Cardiovascular and Metabolic Medicine & Sciences, King’s College London, United Kingdom (K. Theofilatos).

**Keywords:** antibodies, monoclonal, lipidomics, lipoprotein(a), myocardial infarction, proteomics

## Abstract

**BACKGROUND:**

PCSK9 (proprotein convertase subtilisin/kexin type 9) inhibition is a potent cholesterol-lowering strategy. This study examined the effects of PCSK9 monoclonal antibodies (mAbs) and high-intensity statins beyond low-density lipoprotein cholesterol reduction, which are not fully defined, particularly in patients with acute myocardial infarction (MI).

**METHODS:**

Proteomic and lipidomic analyses were conducted on plasma from 265 patients with acute MI from the PACMAN-AMI (Effects of the PCSK9 Antibody Alirocumab on Coronary Atherosclerosis in Patients With Acute Myocardial Infarction) randomized, placebo-controlled PCSK9 mAb trial and 34 patients without MI with hyperlipidemia from the Vienna Lipid Clinic registry, also receiving PCSK9 mAbs.

**RESULTS:**

Discovery proteomics revealed changes in apolipoproteins and increased PCOLCE (procollagen C-endopeptidase enhancer 1) levels in both the PCSK9 mAb and placebo groups after MI. UK Biobank data confirmed PCOLCE and PCSK9 upregulation as associated with statin use. Hepatoma cell experiments demonstrated a dose-dependent PCOLCE induction on statin treatment. Compared with placebo (statins only), PCSK9 mAb therapy resulted in greater reductions in APOB (apolipoprotein B), APOE (apolipoprotein E), APOC2 (apolipoprotein C2), and APOC3 (apolipoprotein C3), as shown by targeted proteomics. Mediation analysis indicated that these changes were largely explained by low-density lipoprotein cholesterol lowering. Lipidomics identified more pronounced reductions in cholesteryl esters, ceramides, sphingomyelins, phosphatidylcholines, triglycerides, and diglycerides in PCSK9 mAb-treated patients with MI. Results were largely consistent in patients without MI. However, levels of LPA (apolipoprotein[a]), the characteristic protein component of lipoprotein(a), remained unchanged in PCSK9 mAb-treated patients with MI, since a rise of LPA was observed in the placebo group post-MI.

**CONCLUSIONS:**

Most apolipoprotein changes after PCSK9 mAb therapy following MI were mediated by low-density lipoprotein cholesterol lowering. Statin use is associated with increased circulating PCOLCE, with hepatoma cell experiments supporting a predominant hepatic origin. Combining PCSK9 mAbs with high-intensity statins mitigates post-MI increases in lipoprotein(a).

**REGISTRATION:**

URL: https://www.clinicaltrials.gov; Unique identifier: NCT03067844.

Positive results from large-scale cardiovascular outcome trials, such as FOURIER (Further Cardiovascular Outcomes Research With PCSK9 Inhibition in Subjects With Elevated Risk)^1^ and ODYSSEY OUTCOMES (Evaluation of Cardiovascular Outcomes After an Acute Coronary Syndrome During Treatment With Alirocumab),^2^ have highlighted the efficacy of monoclonal antibodies (mAbs) targeting PCSK9 (proprotein convertase subtilisin/kexin type 9) in reducing the risk of cardiovascular events. PCSK9 mAbs lower LDL (low-density lipoprotein) cholesterol (LDL-C) by up to 60%, even in patients already receiving statin therapy.^1,2^ Remarkably, within only 12 years after the discovery of PCSK9,^3^ these mAbs were approved by both the European Medicines Agency and the United States Food and Drug Administration. However, -omics data on the effects of PCSK9 mAbs beyond LDL-C lowering are limited in high-risk patient populations.

Despite the additional LDL-C reductions achieved with combination therapy involving statins and PCSK9 mAbs, patients remain at risk for cardiovascular events.^4^ This residual risk is likely due to factors associated with atherosclerotic cardiovascular disease (ASCVD) that are not sufficiently addressed by current therapies. Residual inflammatory risk,^4^ as well as elevated Lp(a) (lipoprotein[a])^5^ and TRLs (triglyceride-rich lipoproteins) and their remnants,^6^ are considered the primary contributors to this residual risk. Supporting this, Mendelian randomization evidence suggests that the cardiovascular benefits of lipid-lowering therapies are not fully explained by reductions in APOB (apolipoprotein B),^7^ the main structural protein of atherogenic lipoproteins (LDL, TRLs, Lp[a]).

As highlighted in a recent review article,^8^ proteomics and lipidomics can advance our understanding of ASCVD. In this study, we integrated proteomics and lipidomics to evaluate the impact of PCSK9 inhibition by mAbs in patients with and without acute myocardial infarction (MI). Understanding how current treatments, including statins and PCSK9 mAbs, affect the plasma proteome and lipidome in patients with MI can expand our knowledge of their overall therapeutic effects beyond LDL-C reduction.

## Methods

### Clinical Cohorts

Two cohorts of patients receiving PCSK9 mAb therapy were included in the study, one consisting of patients with acute MI and another of lipid clinic patients without MI. The PACMAN-AMI trial (Effects of the PCSK9 Antibody Alirocumab on Coronary Atherosclerosis in Patients With Acute Myocardial Infarction)^9^ was approved by all local ethics authorities of the participating centers, with Kantonale Ethikkommission Bern, Switzerland, acting as the lead competent ethics committee. The Vienna Lipid Clinic prospective registry was established in accordance with the Declaration of Helsinki and approved by the Ethics Committee of the Medical University of Vienna (EK no. 1706/2018). Written informed consent was obtained from all participants before their inclusion in the 2 studies. Further details on the cohorts are provided in the Supplemental Material. PACMAN-AMI clinical trial data may be made available from the corresponding author on reasonable request and subject to a specific data-sharing contract.

### UK Biobank

Data from the UKB (UK Biobank) study were analyzed to evaluate associations between statin use and plasma protein levels. The use of statins and any other medications was determined using the UKB baseline (initial assessment visit) treatment/medication code data-field 20003. The list of medications is available online (https://biobank.ctsu.ox.ac.uk/crystal/field.cgi?id=20003). Plasma proteomic data, measured using the antibody-based Olink Explore 3072 platform, are available for a subcohort of 54 219 UKB participants.^10^ Clinical APOA1 (apolipoprotein A1), APOB, and Lp(a) assays were used as orthogonal validation of the corresponding Olink assays. Further details on the UKB participant subsets and data included in this study are provided in the Supplemental Material. Recruitment of UKB participants concluded in 2010, before the approval of PCSK9 mAb therapy in 2015. The present UKB analysis was conducted under research application number 22102.

### Laboratory Measurements

Clinical lipid parameters in PACMAN-AMI patients were measured by the Department of Clinical Chemistry, University of Zurich, Switzerland. Clinical lipid parameters in Vienna Lipid Clinic patients were measured by the Department of Laboratory Medicine, Medical University of Vienna, Austria. Mass spectrometric data sets were acquired in-house. Detailed information regarding the hepatoma cell experiments, immunoblotting, and mass spectrometry (MS) analyses is available in the Supplemental Material. MS proteomics data from the hepatoma cell experiments have been deposited to the ProteomeXchange Consortium via the PRIDE partner repository with the digital object identifier 10.6019/PXD069776.

### Statistical Analysis

The statistical analysis of MS data is presented together with acquisition parameters in the Supplemental Material. Analysis of UKB data was performed using R (v4.0.2). After excluding participants who had withdrawn and those with ≥30% missing values in Olink proteomic quantification, 44 395 individuals were retained for Olink analysis. Olink proteins with ≥30% missing values, namely GLIPR1 (glioma pathogenesis-related protein 1), NPM1 (nucleophosmin), and PCOLCE (procollagen C-endopeptidase enhancer 1), were initially dropped. Missing Olink values were KNN-imputed (K=10) using the *impute* package (v1.80.0). Clinical APOA1, APOB, and Lp(a) assay values (Supplemental Material) were log_2_ transformed, and no data imputation was performed for clinical assays. Statin versus no statin comparisons for Olink and clinical assay data were conducted in 3 sets of participants, retaining only individuals with complete clinical assay data: (1) all available participants after the above filtering steps, including those receiving other medications in addition to statins; (2) a subset excluding participants on any other medication; and (3) a subset restricted to participants with quantifiable PCOLCE levels and no other medications. Although our MS-based proteomics analysis in the PACMAN-AMI cohort only had 8% missing PCOLCE data, 63.1% of PCOLCE Olink data failed quality control (see Table S3 of Sun et al^10^), leading to its exclusion in the first 2 analyses based on the 30% missing value threshold. Comparisons between statin users and nonusers in Olink and clinical assays were performed by applying the eBayes method from the *limma* package, adjusting for age (UKB data-field 21022) and sex (UKB data-field 31) as covariates. For visualization, the significance threshold for comparisons between statin users and nonusers was set at an adjusted *P* value of 5×10^−8^.

Mediation analysis was performed to decompose the PCSK9 mAb effect on apolipoprotein and lipid parameters into direct and LDL-C-mediated indirect components. The average causal mediation effect (ACME), average direct effect, and the total effect were estimated using the *mediation* package (v4.5.0). Parameters were selected if they showed larger 4-week decreases with alirocumab versus placebo using a Wilcoxon rank-sum test with Benjamini-Hochberg-adjusted *P*<0.05. LDL-C itself was excluded. For each selected parameter, 2 linear regression models were fit: (1) the mediator model: LDL-C change=treatment; and (2) the outcome model: apolipoprotein or lipid parameter change=treatment+LDL-C change. Changes were defined as log_2_-fold changes between the 4-week and baseline time points, and effects were back-transformed to the linear scale and expressed as percent differences for interpretability. ACME, average direct effect, and total effect were estimated with the mediate function using nonparametric bootstrapping (1000 simulations). The proportion mediated by LDL-C was calculated as ACME divided by the total effect for each parameter. To facilitate interpretability in cases where the directionality of effects differed (ie, opposite signs for ACME and average direct effect), proportions exceeding 100% were capped at 100%. To assess robustness to potential pretreatment confounders, we conducted a sensitivity analysis, adjusting for age, sex, baseline statin use (yes/no), and baseline LDL-C in both the mediator and outcome models. *P* values were adjusted using the Benjamini-Hochberg method across the parameters that entered the mediation step. The criterion for significant mediation was ACME with an adjusted *P*<0.05.

All statistical analyses were 2-sided. Data visualization was performed using the *ggplot2* (v3.5.0) and *patchwork* (v1.2.0) packages. References for the R packages used can be found in the Supplemental Material.

## Results

### Discovery Proteomics in Patients With Acute MI

In the PACMAN-AMI trial, 300 patients with acute MI were randomized to receive either the PCSK9 mAb alirocumab (n=148) or placebo (n=152) alongside high-intensity statin therapy. The clinical characteristics of the 265 patients who completed the 52-week follow-up with serial-blood samples collected at baseline, 4 weeks, and 52 weeks, are presented in Table 1. Notably, most patients were statin-naive at baseline, with only 11.5% in the PCSK9 mAb group and 14.2% in the placebo group having received prior statin therapy. To mitigate potential confounding effects of heparin^11^ on baseline measurements, additional samples (n=85) were collected 24 hours after percutaneous coronary intervention from the PACMAN-AMI Bern subcohort (Table S1).^9^

**Table 1.**
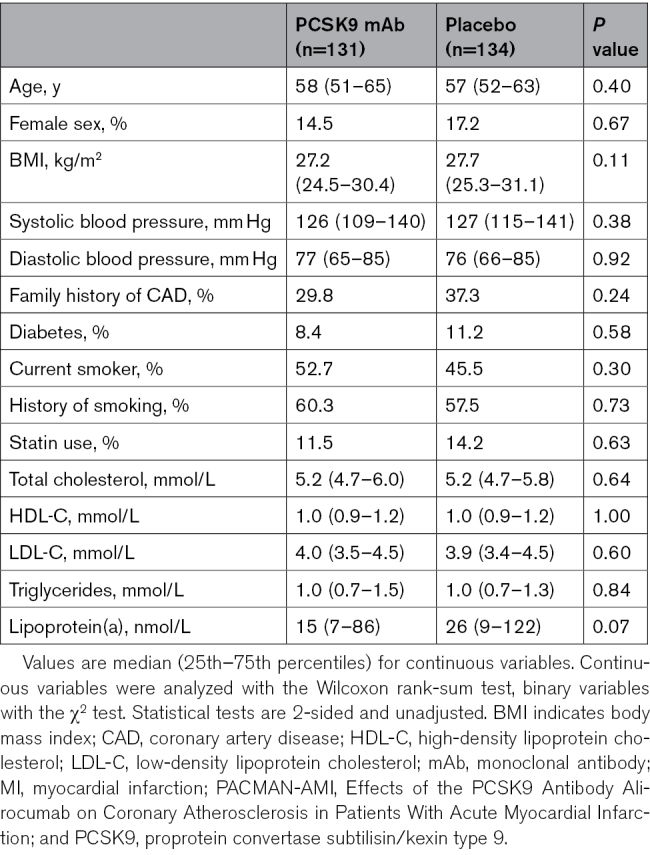
Baseline Characteristics of 265 Patients With Acute MI From the PACMAN-AMI Trial Who Completed the 52-Week Follow-Up

**Figure 1.**
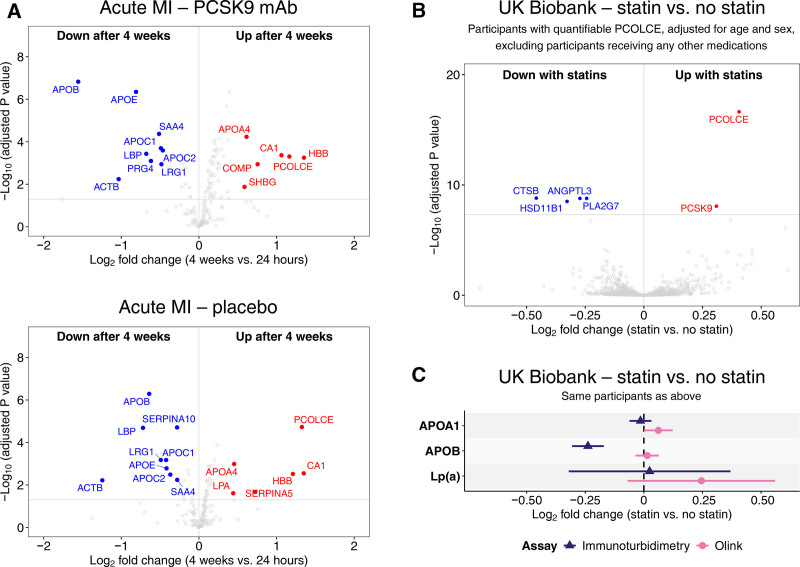
**Plasma proteomic changes with PCSK9 (proprotein convertase subtilisin/kexin type 9) monoclonal antibody (mAb) or placebo in patients with acute myocardial infarction (MI) and associations with statin use in UK Biobank participants. A**, Four-week proteomic changes from the 24-hour post-percutaneous coronary intervention time point with PCSK9 mAb (**top**) or placebo (**bottom**) treatment in a subset of 44 patients with acute MI, quantified by mass spectrometry. **B**, Differences in Olink-based plasma protein levels between statin users (n=89) and statin-naive UK Biobank participants (n=3035) with quantifiable PCOLCE (procollagen C-endopeptidase enhancer 1) levels, excluding participants receiving any other medications besides statins. Significance threshold was set at adjusted *P*=5×10^−8^. **C**, Comparison of immunoturbidimetric (dark blue) and Olink (pink) assays measuring APOA1 (apolipoprotein A1), APOB (apolipoprotein B), and Lp(a) (lipoprotein[a]) in the same UK Biobank participants as shown in **B**. Error bars indicate the 95% CI. Olink values are reported as the LPA (apolipoprotein[a]) protein, while the immunoturbidimetric assay measures the Lp(a) particle. In **A** and **B**, *P* values were adjusted for multiple testing using the Benjamini-Hochberg method. ACTB indicates actin, cytoplasmic 1; ANGPTL3, angiopoietin-related protein 3; CA1, carbonic anhydrase 1; COMP, cartilage oligomeric matrix protein; CTSB, cathepsin B; HBB, hemoglobin subunit beta; HSD11B1, 11-beta-hydroxysteroid dehydrogenase 1; LBP, lipopolysaccharide-binding protein; LRG1, leucine-rich alpha-2-glycoprotein; PLA2G7, platelet-activating factor acetylhydrolase; PRG4, proteoglycan 4; SAA4, serum amyloid A-4 protein; SERPINA5, plasma serine protease inhibitor; SERPINA10, protein Z-dependent protease inhibitor; and SHBG, sex hormone-binding globulin.

Discovery proteomics by MS revealed similar sets of dysregulated plasma proteins in both the PCSK9 mAb and placebo groups (Figure 1A; Table S2). At 4 weeks after MI, compared with 24 hours, both groups showed a reduction in acute-phase proteins (SAA4 [serum amyloid A-4 protein]; LBP [lipopolysaccharide-binding protein]; and LRG1 [leucine-rich alpha-2-glycoprotein]) alongside increases in erythrocyte proteins (CA1 [carbonic anhydrase 1] and HBB [hemoglobin subunit beta]) and procollagen C-endopeptidase enhancer 1 (PCOLCE).

Interestingly, ECM (extracellular matrix) components, such as COMP (cartilage oligomeric matrix protein) and PRG4 (proteoglycan 4), were differentially regulated in the PCSK9 mAb group but not in the placebo group. Treatment with PCSK9 mAb also led to greater reductions in APOB and APOE (apolipoprotein E) levels compared with placebo, while changes in APOC1 (apolipoprotein C1), APOC2 (apolipoprotein C2), and APOA4 (apolipoprotein A4) were similar between groups. LPA (apolipoprotein[a]), the characteristic protein component of Lp(a), showed a significant increase only in the placebo group, with no rise observed in the PCSK9 mAb group.

### Analysis of Statin Effects Using UKB Data

To distinguish the effects of new-onset high-intensity statin therapy on plasma protein levels from those of the acute MI itself, we interrogated the Olink proteomics data from the UKB study. We first compared protein levels between statin users and statin-naive participants, regardless of other medications (n=30 632; Figure S1; Table S3). Statin users showed significantly lower levels of APOC1, APOD (apolipoprotein D), APOE, and APOM (apolipoprotein M), whereas levels of LPA were higher.

To isolate statin-specific effects, we excluded participants on concomitant medications, yielding a subset of 12 014 individuals (Figure S2; Table S4). In this group, PCSK9 was the most significantly upregulated protein in statin users. Conversely, PLA2G7 (platelet-activating factor acetylhydrolase), ANGPTL3 (angiopoietin-related protein 3), CTSB (cathepsin B), and HSD11B1 (11-beta-hydroxysteroid dehydrogenase 1) were significantly reduced in statin users compared with statin-naive participants.

To validate the PCOLCE increase observed in both acute MI groups, we analyzed a subset of statin-treated UKB participants with available quantitative PCOLCE measurements by Olink and no other medications (n=3124; Figure 1B; Table S5). In this cohort, PCOLCE was the most significantly upregulated protein, confirming the MS-based discovery proteomics results. Statin treatment also resulted in a compensatory increase in PCSK9. Downregulated proteins were consistent with those identified in the broader UKB analyses (Figures S1 and S2).

Among apolipoproteins with corresponding clinical measurements (APOA1, APOB, and Lp[a]), the Olink assay failed to detect the statin-induced reduction in APOB (adjusted *P*=1.6×10^−11^; Figure 1C). A discrepancy between clinical and Olink measurements of APOB, as well as discordant Lp(a) quantification (Figure S3), was observed across the UKB data set.

### Analysis of Statin Effects Using LPA-Expressing Human Hepatoma Cells

**Figure 2.**
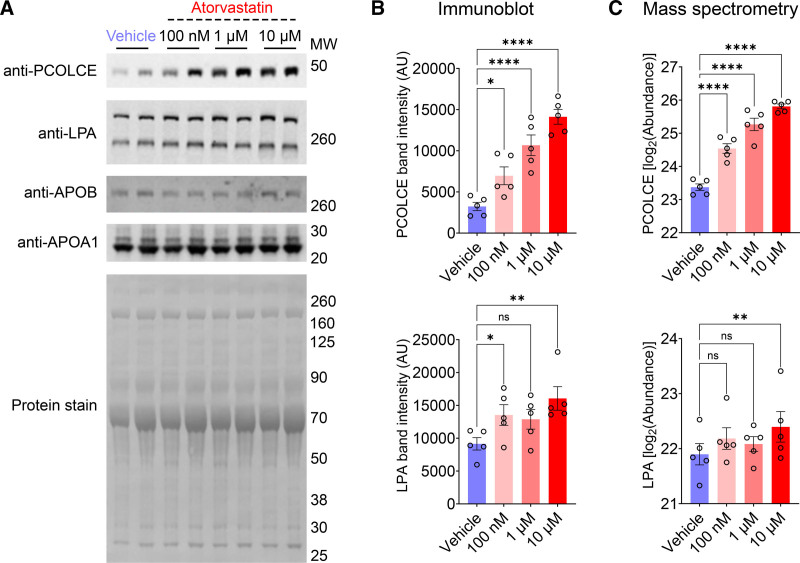
**Protein responses in conditioned media of atorvastatin-treated hepatoma cells.** Medium of HepG2 cells expressing LPA (apolipoprotein[a]) was conditioned with the indicated concentrations of atorvastatin for 10 days. Vehicle was dimethyl sulfoxide. **A**, Immunoblots for indicated proteins. **B**, PCOLCE (procollagen C-endopeptidase enhancer 1) (**top**) and LPA (**bottom**) densitometric quantification associated with the blots shown in **A. C**, PCOLCE (**top**) and LPA (**bottom**) quantification by mass spectrometry. AU indicates arbitrary unit; MW, molecular weight; and ns, not significant. Significant *P* values from 2-way repeated-measures ANOVA are indicated by asterisks: ****P<0.0001; **P value between 0.001 and 0.01; *P value between 0.01 and 0.05.

To further investigate the rise in circulating LPA and PCOLCE in statin users, HepG2 cells stably expressing LPA were treated with atorvastatin for 10 days. Escalating concentrations resulted in higher intracellular levels of HMG-CoA (3-hydroxy-3-methylglutaryl-coenzyme A) reductase detected by MS (Figure S4), confirming on-target activity. In the conditioned media, LPA increased modestly and PCOLCE markedly in response to atorvastatin, as shown by immunoblotting (Figure 2A and 2B) and MS (Figure 2C). PCOLCE was among the most upregulated proteins in the statin-conditioned media (data not shown). APOA1 and APOB remained unchanged (Figure S5).

### Validation by Targeted Proteomics in Patients With Acute MI

To validate our apolipoprotein findings by discovery proteomics, we used targeted MS to quantify 14 apolipoproteins in 265 patients with acute MI across all time points. Longitudinal data confirmed that the lipid-lowering effect of PCSK9 mAb treatment peaked at 4 weeks (Figure S6), aligning with the time point selected for discovery proteomics and lipidomics.

Strong correlations were observed between clinical lipid measures and MS-based apolipoprotein quantification (Figure S7), including clinical Lp(a) and LPA (Spearman *r*=1.0), LDL-C and APOB (*r*=0.9), and triglycerides and APOC2, APOC3 (apolipoprotein C3), and APOE (*r*=0.5–0.7).

### Mediation Analysis of PCSK9 mAb Effects on Clinical Lipids and Apolipoproteins

**Figure 3.**
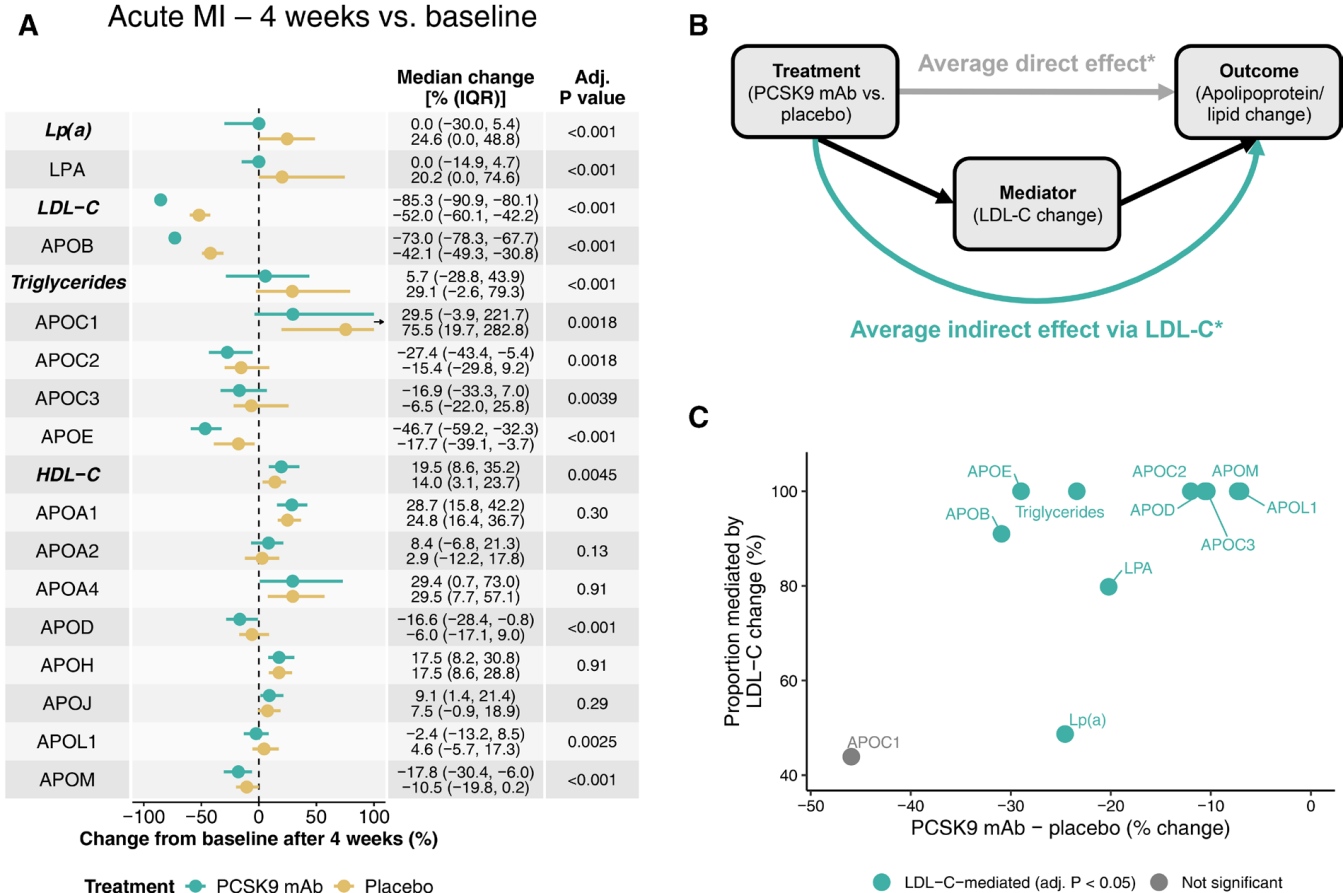
**Apolipoprotein and lipid changes with PCSK9 (proprotein convertase subtilisin/kexin type 9) monoclonal antibody (mAb) or placebo treatment. A**, Four-week changes from baseline in 260 patients with acute myocardial infarction (MI; PACMAN-AMI [Effects of the PCSK9 Antibody Alirocumab on Coronary Atherosclerosis in Patients With Acute Myocardial Infarction]) comparing PCSK9 mAb (green) and placebo (gold). The black arrow next to the APOC1 (apolipoprotein C1) graph indicates cropped interquartile range (IQR) indicators. Changes are median percent changes (25th, 75th percentiles). *P* values are from the Wilcoxon rank-sum test for between-treatment comparisons. **B**, Mediation diagram for the analysis conducted on data displayed in **A**, illustrating the distinction between direct treatment effects and indirect effects mediated through low-density lipoprotein cholesterol (LDL-C) change. The analysis included only parameters with significantly greater reductions in the PCSK9 mAb arm compared with placebo (adj. [adjusted] *P*<0.05, **A**). Results of the analysis are shown in **C. C**, Scatter plot highlighting the extent to which reductions in apolipoprotein and lipid parameters are mediated through changes in LDL-C levels. Parameters with significant mediation by LDL-C (adj. *P*<0.05) are marked in green. All *P* values were adjusted for multiple testing using the Benjamini-Hochberg method. HDL-C indicates high-density lipoprotein cholesterol; LPA, apolipoprotein(a); and Lp(a), lipoprotein(a). *Direct and indirect effects for the analyzed apolipoprotein and lipid parameters are shown in Table 2.

We analyzed the effects of PCSK9 mAb treatment on apolipoprotein and clinical lipid concentrations from baseline to 4 weeks (Figure 3A). Compared with placebo, PCSK9 mAb treatment resulted in significantly greater reductions in LDL-C (−85.3% versus −52.0%) and APOB (−73.0% versus −42.1%) levels. Larger reductions were also observed in triglyceride-associated apolipoproteins (APOE, APOC2, APOC3), as well as APOD, APOL1 (apolipoprotein L1), and APOM.

**Table 2.**
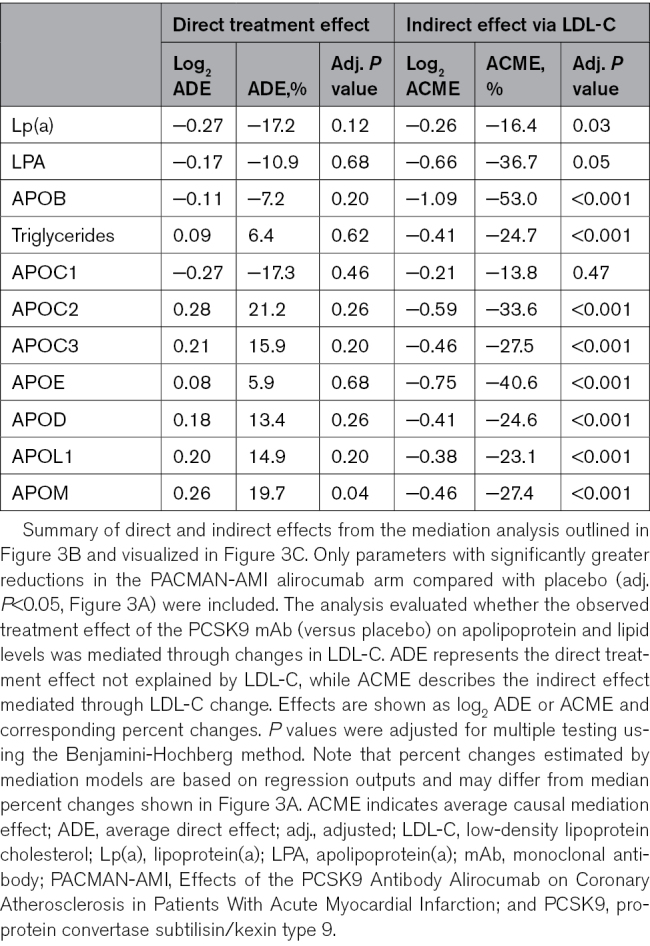
Mediation of PCSK9 mAb Effects on Apolipoproteins and Clinical Lipids via LDL-C Change

Mediation analysis (Figure 3B; Table 2) revealed that most of the lipid and apolipoprotein reductions associated with PCSK9 mAb treatment were entirely mediated by LDL-C lowering. APOB, LPA, and clinical Lp(a) were only partially mediated by changes in LDL-C (Figure 3C). Inclusion of the covariates age, sex, baseline statin status, and baseline LDL-C in a sensitivity analysis did not materially affect the estimated mediation effects (Figure S8; Table S6). These findings were validated by comparing apolipoprotein and lipid measurements at the 24-hour and 4-week time points (Figure S9).

### Comparison of PCSK9 mAb Treatment Effect in Patients With Acute MI and Patients Without MI

**Figure 4.**
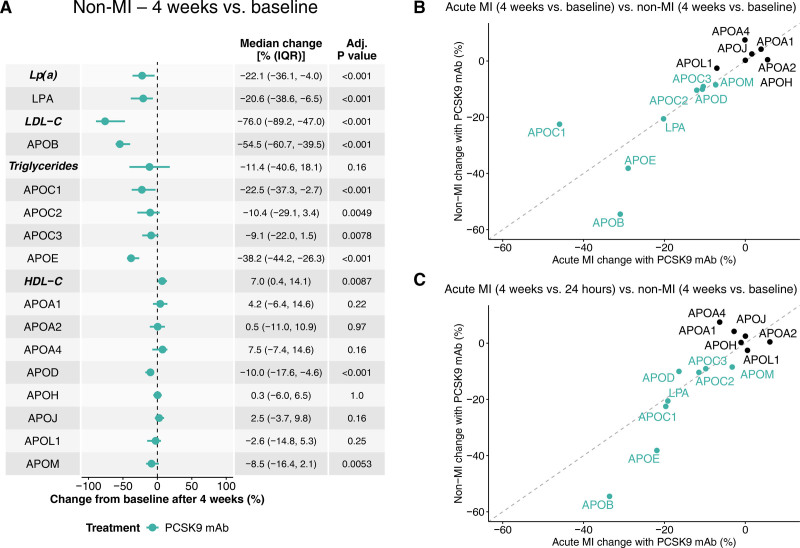
**Comparative changes in apolipoprotein levels with PCSK9 (proprotein convertase subtilisin/kexin type 9) monoclonal antibody (mAb) treatment in patients with acute myocardial infarction (MI) and patients without MI. A**, Four-week changes from baseline in 34 patients without MI (Vienna Lipid Clinic) after PCSK9 mAb treatment. Changes are median percent changes (25th, 75th percentiles). *P* values are from the Wilcoxon signed-rank test for paired comparisons between baseline and 4 weeks. All *P* values were adjusted (adj.) for multiple testing using the Benjamini-Hochberg method. **B** and **C**, Correlation of changes in apolipoprotein levels between the acute MI and non-MI cohorts, with both cohorts assessed for changes from baseline (**B**) or 24 hours (**C**) after 4 weeks of treatment. Changes are median percent changes, with the acute MI values representing the difference between the median percent change with PCSK9 mAb and the median percent change with placebo. Green color denotes apolipoproteins that exhibit significant changes in response to 4-week PCSK9 mAb treatment in both the acute MI and non-MI cohorts. HDL-C indicates high-density lipoprotein cholesterol; IQR, interquartile range; LDL-C, low-density lipoprotein cholesterol; LPA, apolipoprotein(a); and Lp(a), lipoprotein(a).

To validate our findings in patients with acute MI, we repeated the MS comparison in patients without MI (N=34; Figure 4A) treated for severe hypercholesterolemia in the Vienna Lipid Clinic prospective registry (Table S7). We then compared the effects of PCSK9 mAb therapy in acute MI and patients without MI at the 4-week time point. Patients with acute MI experienced greater median reductions in LDL-C (−85.3% versus −76.0%) and APOB (−73.0% versus −54.5%) after 4 weeks compared with patients without MI (Figure 4B and 4C).

Besides APOB, decreases in triglyceride-associated apolipoproteins (APOE, APOC2, APOC3), as well as APOD and APOM, were observed in both groups after PCSK9 mAb treatment. In the acute MI group, baseline levels of triglycerides and APOC1 were likely impacted by heparin administration, which affects lipoprotein lipase activity.^11^ Unlike the baseline-to-week-4 comparison (Figure 3A), a reduction in APOC1 was seen in the PACMAN-AMI Bern subcohort between the 24-hour and 4-week time points (Figure S9), consistent with the findings in patients without MI (Figure 4A).

APOA1, APOA4, and APOH (beta-2-glycoprotein 1) increased after acute MI (Figure 3A), changes not seen in the non-MI cohort. Additionally, while patients without MI showed reductions in Lp(a) and LPA (−22.1% and −20.6%, respectively), no LPA reduction was observed in the acute MI group after PCSK9 mAb therapy.

### Correlation of Baseline Protein and Lipid Levels With Treatment Responses

APOB, APOE, and LPA exhibited the most substantial and consistent reductions after 4 weeks of PCSK9 mAb treatment (Figure 4A). To explore predictors of treatment response, we performed Spearman correlation analyses between baseline protein or lipid levels and treatment-induced changes in these apolipoproteins (Figure S10).

Baseline plasma PCSK9 levels showed no correlation with changes in APOB, APOE, or LPA. In contrast, baseline LDL-C and clinical triglyceride levels were inversely correlated with changes in APOB and APOE, respectively. Even stronger inverse correlations were observed for baseline APOB and APOE, indicating that higher baseline levels were associated with greater reductions after treatment.

Baseline clinical Lp(a) and LPA by MS were positively correlated with LPA change, with significantly stronger associations in the placebo group. These, along with the APOB/LDL-C relationship, were the only correlations showing a differential treatment effect based on *Z* tests. Similar, but less pronounced patterns were observed when analyzing relative percent changes (Figure S11).

### Corresponding Lipidomic Changes in Patients With Acute MI

**Figure 5.**
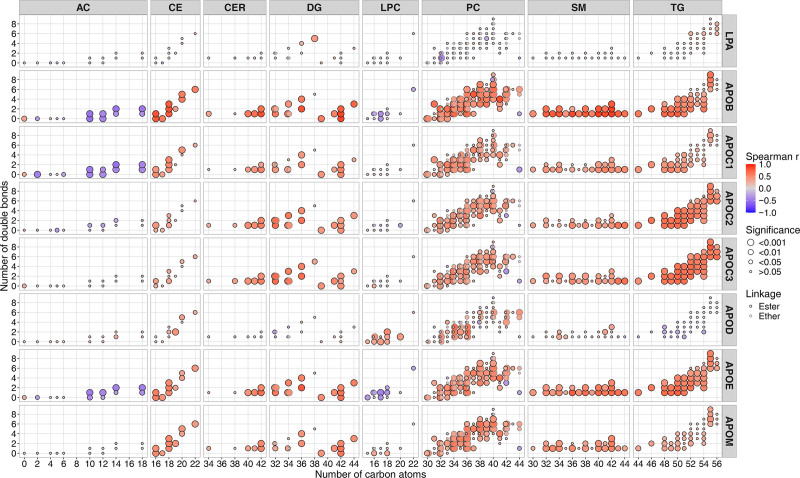
**Correlation between plasma lipid species and apolipoproteins in patients with acute myocardial infarction (MI).** Correlations are shown as filled circles with a border. The number of carbon atoms is shown on the *x* axes, and the number of double bonds on the *y* axes. Circle fill color represents the Spearman correlation coefficient (*r*) between individual lipids and apolipoproteins, circle size represents the categorized significance level, and circle border color indicates the linkage type. Some lipids have equal numbers of carbon atoms and double bonds but differ in the linkage of their alk(en)yl chains. In these cases, ester-linked lipids are pulled to the left and ether-linked lipids to the right for better visibility. Correlations were calculated between 193 individual lipids and 8 apolipoproteins, measured across 70 samples. All *P* values were adjusted for multiple testing using the Benjamini-Hochberg method. AC indicates acylcarnitine; CE, cholesteryl ester; CER, ceramide; DG, diglyceride; LPA, apolipoprotein(a); LPC, lysophosphatidylcholine; PC, phosphatidylcholine; SM, sphingomyelin; and TG, triglyceride.

To complement our apolipoprotein proteomics approach, we analyzed lipidomic changes by comparing plasma levels at 24 hours and 4 weeks after MI. A total of 193 lipid species across 8 classes were consistently quantified. Correlations between these lipid species and apolipoproteins significantly affected by PCSK9 mAb treatment are presented in Figure 5.

Except for LPA and APOD, the altered apolipoproteins showed strong positive associations with cholesteryl esters, ceramides, sphingomyelins, phosphatidylcholines, triglycerides, and diglycerides. APOD was positively associated with lysophosphatidylcholines, while LPA showed minimal correlation with most lipid species.

**Figure 6.**
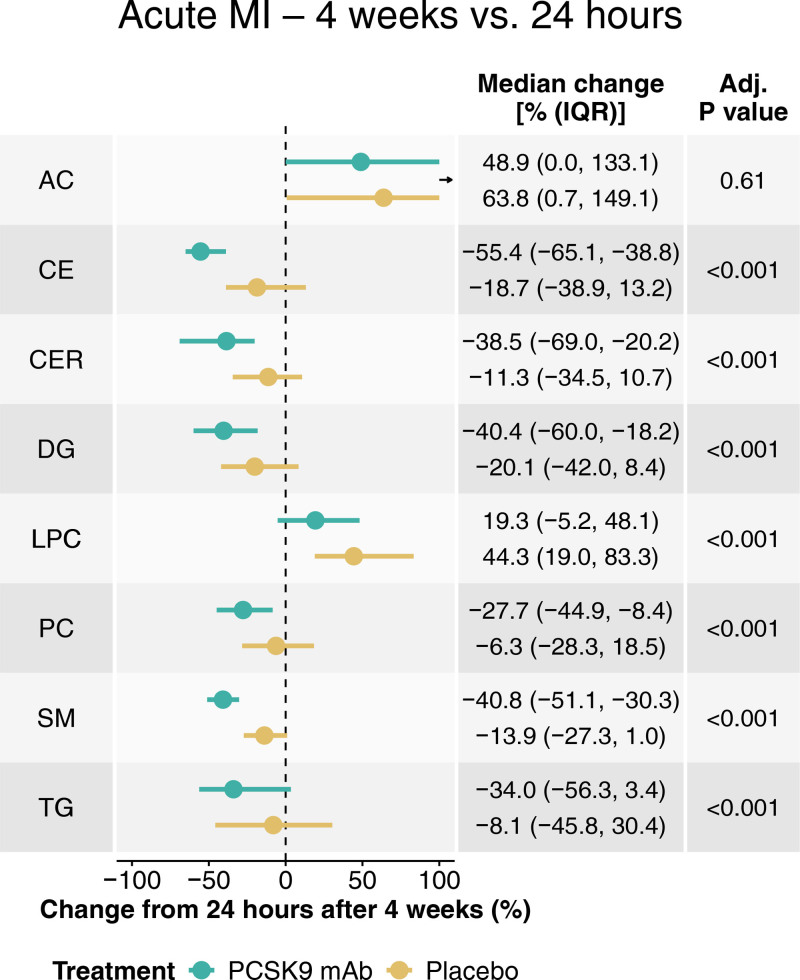
**Plasma lipidomic changes with PCSK9 (proprotein convertase subtilisin/kexin type 9) monoclonal antibody (mAb) or placebo in patients with acute myocardial infarction (MI).** Four-week lipidomic changes from the 24-hour post-percutaneous coronary intervention time point with PCSK9 mAb (green) or placebo (gold) treatment in a subset of 35 patients with acute MI. 193 lipids from 8 lipid classes were consistently quantified, with median percent changes (25th, 75th percentiles) per class displayed. *P* values are from the Wilcoxon rank-sum test for between-treatment comparisons and were adjusted (adj.) for multiple testing using the Benjamini-Hochberg method. Number of patients per treatment group: PCSK9 mAb (20), placebo (15). Number of measured individual lipids per class: acylcarnitine (AC; 13), cholesteryl ester (CE; 10), ceramide (CER; 6), diglyceride (DG; 15), lysophosphatidylcholine (LPC; 11), phosphatidylcholine (PC; 79), sphingomyelin (SM; 22), triglyceride (TG; 37). The black arrow next to the AC graph indicates cropped interquartile range (IQR) indicators.

Acylcarnitines increased after MI in both the PCSK9 mAb and placebo groups (Figure 6; Table S8). The greatest reductions under PCSK9 mAb therapy, compared with placebo, were observed for cholesteryl esters, followed by ceramides, sphingomyelins, phosphatidylcholines, triglycerides, and diglycerides. Notably, PCSK9 mAb therapy also attenuated the post-MI rise in lysophosphatidylcholines.

## Discussion

We combined proteomics and lipidomics to investigate the broader biological effects of PCSK9 mAbs and statins, revealing 3 key findings. First, statin therapy was associated with effects beyond LDL-C lowering, most prominently an increase in the collagen modulator PCOLCE, which rose similarly in both treatment arms of the PACMAN-AMI trial. This was validated by UKB data and through in vitro experiments involving statin treatment of hepatoma cells, the latter implicating the liver as a likely source of elevated circulating PCOLCE levels. Second, PCSK9 mAb therapy reduced triglyceride-associated apolipoproteins APOE, APOC2, and APOC3. The effect was consistent across 2 independent cohorts with and without MI and was largely mediated by LDL-C reduction. Third, a rise in LPA was observed in placebo-treated patients after MI, correlating with baseline LPA levels, but this was not seen in the PCSK9 mAb group. A modest rise in LPA secretion was also seen in statin-treated hepatoma cells.

### Hepatocyte Contribution to Statin-Induced PCOLCE Increase

Discovery proteomics in the acute MI cohort revealed an increase in PCOLCE levels after 4 weeks, with similar effect sizes in both the PCSK9 mAb and placebo groups. PCOLCE enhances the activity of procollagen C-endopeptidase, an enzyme that cleaves procollagen to form mature collagen. Statin therapy, which promotes delipidation of atherosclerotic lesions^12^ and leads to fibrotic remodeling and plaque calcification,^13^ has been associated with increases in PCOLCE.^14,15^ Supporting this association, we demonstrated higher PCOLCE levels in statin users compared with nonusers in the UKB. While direct evidence for circulating PCOLCE levels and cardiovascular end points is limited, Kolossváry et al linked higher circulating PCOLCE levels on statin treatment to greater reductions in noncalcified coronary plaque volume.^15^ In their study, however, the cellular origin of circulating PCOLCE remained unclear. In our experiments, atorvastatin treatment led to a pronounced, dose-dependent induction of PCOLCE in human hepatoma cells. Taken together, these results point to a statin-associated increase in circulating PCOLCE, likely originating from the liver. By promoting collagen maturation, PCOLCE supports tissue remodeling and contributes to fibrosis.^16^ Therefore, post-MI cardiac remodeling may also contribute to the rise in PCOLCE observed in the acute MI cohort.

### Wider Associations of Statin Use and Protein Levels in UKB Participants

Given the initiation of high-intensity statin therapy after MI, we investigated broader statin effects using UKB data. Comparing statin users with statin-naive participants confirmed that statins elevate PCSK9 levels.^17^ This underscores the synergy with PCSK9 mAbs, which counteract this statin-induced compensatory increase in PCSK9, thereby enhancing their LDL-C-lowering effect.

Statin users also showed lower levels of ANGPTL3, a lipoprotein lipase inhibitor and established ASCVD risk factor, potentially contributing to greater triglyceride reductions. This is consistent with Olink proteomic data from the REPRIEVE (Randomized Trial to Prevent Vascular Events in Human Immunodeficiency Virus) trial showing lower circulating ANGPTL3 with statin therapy. In the latter study, higher ANGPTL3 levels were independently associated with an increased risk of major adverse cardiovascular events.^18^ In line with ANGPTL3 being pursued as a target to lower remnant cholesterol, Balling et al^19^ modeled ASCVD risk using data from the Copenhagen General Population Study and found that risk reductions scaled with the extent of remnant cholesterol lowering.

Furthermore, our UKB analysis revealed that statin use was associated with reduced levels of PLA2G7 and CTSB. Although the phospholipase PLA2G7 has been linked to ASCVD risk, it remains unclear whether this reflects its proinflammatory activity or its correlation with LDL levels.^20^ In our previous proteomic analyses of carotid plaques,^21,22^ macrophage-derived lysosomal proteases, including CTSB, consistently emerged as a prominent component of the inflammatory signature in symptomatic patients.

Lastly, in the UKB, the Olink assay failed to detect the expected reduction in APOB levels in statin users. This finding highlights known cross-platform discrepancies in apolipoprotein quantification.^23^

### Triglyceride-Associated Apolipoprotein Reduction Is Explained by LDL-C Lowering

Unlike affinity-based assays, targeted MS offers direct peptide quantification using stable isotope-labeled internal standards, eliminating epitope effects. Given the broad apolipoprotein dysregulation in the PACMAN-AMI cohort, we applied targeted MS and found that PCSK9 mAb treatment reduced APOE, APOC2, and APOC3 in both the MI and non-MI cohorts. These predominantly triglyceride-related apolipoproteins have been consistently associated with cardiovascular risk,^24,25^ and similar changes have been reported in healthy volunteers,^26,27^ hyperlipidemic patients without MI,^28^ and individuals with recent acute coronary syndrome.^29^

Importantly, our analysis showed that these reductions were mediated by LDL-C lowering. LDL-C is a composite measure comprising the cholesterol content of LDL, IDL (intermediate-density lipoprotein), and Lp(a) particles.^30^ The observed apolipoprotein changes primarily reflect on-target reductions in LDL and IDL particles rather than direct, independent effects on TRLs. This interpretation is consistent with 2 kinetic studies in healthy, normolipidemic volunteers demonstrating increased LDL and IDL catabolism and reduced LDL production with PCSK9 mAb therapy.^26,27^ The larger of the 2 studies (N=81)^27^ also reported enhanced VLDL (very low-density lipoprotein) catabolism, particularly under concomitant statin therapy. Similarly, a post hoc analysis of 3 placebo-controlled phase 2 trials including hypercholesterolemic patients (N=171) showed reductions not only in LDL-C and its subfractions but also in VLDL-C, IDL-C, APOC2, and APOC3 levels, again with larger effects under combination therapy.^31^

Although our lipidomics data showed marked reductions in triglycerides and diglycerides, these species are also abundant in LDL and IDL, supporting a predominant contribution of enhanced LDL/IDL clearance with only a minor TRL component. As LDL-C integrates information from multiple particle types, apolipoprotein profiling could complement the traditional lipid panel. In an ODYSSEY OUTCOMES substudy, a 9-plex apolipoprotein panel outperformed the conventional lipid panel for event prediction, and combined models performed best, with additional exploration of apolipoproteins for guiding PCSK9 mAb treatment selection.^32^ In our study, higher baseline APOB and APOE levels were associated with greater on-treatment reductions, highlighting the potential value of measuring apolipoproteins in secondary prevention.

### ECM and Acute-Phase Protein Dysregulation After Acute MI

Beyond apolipoproteins and PCOLCE, other ECM proteins, such as COMP and PRG4, were differentially regulated in the acute MI cohort. PRG4 (lubricin), a glycoprotein primarily recognized for reducing friction between cartilage surfaces in joints, was reduced only in the PCSK9 mAb group. Notably, PRG4 has been linked to the osteogenic transformation of valve interstitial cells,^33^ a central process in aortic stenosis, which is currently under investigation as a potential treatment indication for PCSK9 inhibitors.^34^ Whether ECM protein differences with PCSK9 mAb therapy reflect distinct healing processes compared with statin therapy alone warrants future investigation.

Both treatment groups showed downregulation of acute-phase proteins, including LBP, LRG1, and SAA4, consistent with the resolution of inflammation after MI. Beyond acute-phase proteins, apolipoproteins showed the greatest reductions, with PCSK9 mAbs exerting a stronger effect than high-intensity statins. In contrast, APOA4 increased at 4 weeks in both groups, aligning with our previous observation of APOA4 upregulation during cardiac ECM remodeling after ischemia/reperfusion injury.^35^

### PCSK9 mAbs Attenuate Post-MI Rise in LPA and Lp(a)

Lp(a) is a major contributor to residual lipid risk, with UKB data showing that LPA-containing particles are more atherogenic than LDL on a per-particle basis.^36^ PCSK9 mAbs typically reduce Lp(a) by 20% to 30%,^37^ but this effect was not apparent in patients with acute MI. Previously, we reported a rise in Lp(a) from the immediate post-MI period to 1 year in young patients receiving statins, with the magnitude of the rise dependent on baseline Lp(a) levels.^38^ In the current study, we observed a similar increase from the post-MI period to 4 weeks in the placebo group, but not in the PCSK9 mAb group. The apparent rise in Lp(a) may be explained by lower baseline levels immediately after MI. It has been suggested that Lp(a) can act as an acute-phase protein, possibly being consumed during thrombus formation and tissue repair.^39^ As inflammation resolves, Lp(a) levels may return to pre-MI levels. However, some studies dispute an acute-phase role of Lp(a), as its post-MI trajectory differs from that of high-sensitivity C-reactive protein.^40^ Residual low-grade inflammation, particularly involving interleukin-6, which induces Lp(a),^41^ may also contribute to elevated Lp(a) after MI.

Almost 90% of patients with acute MI were statin-naive at baseline. Post-MI initiation of high-intensity statin therapy may raise Lp(a) levels,^42^ consistent with our in vitro data showing modest LPA induction in conditioned media of atorvastatin-treated hepatoma cells. Other studies, including our MS data from participants of the ASCOT (Anglo-Scandinavian Cardiac Outcomes Trial) study receiving low-dose atorvastatin,^25^ found no significant changes.^43^ In the UKB, statin use was associated with a rise in LPA levels in the overall statin versus no statin comparison. In the subcohort of participants with detectable PCOLCE, the trend was similar but not statistically significant, likely due to the smaller sample size, though this effect was minimal relative to the cardiovascular benefits of LDL-C lowering by statin treatment.^44^ However, confirmation of high intensity statin-induced increases in Lp(a) would strengthen the rationale for prioritising patients with high Lp(a) for PCSK9 inhibitor therapy. Divergent findings on the effects of statins on Lp(a) could point to interindividual variability, potentially influenced by underexplored factors, such as LPA posttranslational modifications.^45^ Our UKB comparison also revealed discrepancies between Olink and clinical assays for Lp(a). By contrast, our MS-based apolipoprotein quantification exhibited a near-perfect correlation with clinically measured Lp(a), detecting comparable fold changes in both treatment arms of PACMAN-AMI.

### Limitations

Participants in the PACMAN-AMI trial were enrolled at European centers, limiting ethnic diversity. Large-scale PCSK9 mAb outcome trials such as ODYSSEY OUTCOMES showed no significant interaction between treatment effect on primary end points and ethnicity,^2^ although non-White populations were underrepresented in these studies. Genetic variation may have a greater influence through effects on baseline lipid and apolipoprotein levels rather than differential treatment responses, as exemplified by well-established ethnic differences in Lp(a) concentrations^46^ and by ancestry-specific protein quantitative trait loci (eg, *APOL1*).^47^

A key limitation of -omics analyses in the context of acute MI is the potential for confounding effects from the ischemic event and clinical interventions, such as heparin administration. To mitigate this, a 24-hour post-MI sampling time point was included in a subcohort, along with validation in an independent cohort of patients without acute MI.

### Conclusions

This study represents the largest serial-blood multiomics analysis to date examining PCSK9 mAb therapy in the secondary prevention of ASCVD. In both PACMAN-AMI and the UKB, we confirm a statin-associated increase in the collagen modulator PCOLCE, with in vitro data implicating the liver as a potential source of the elevated circulating PCOLCE. Given that the liver is both the principal site of statin action and a major secretory organ, hepatic release represents the most biologically plausible explanation, although contributions from other sources, that is, cardiac extracellular matrix remodeling and atherosclerotic plaques, remain to be clarified. The combination of PCSK9 mAbs and statins counteracts the statin-induced increase in PCSK9 and achieves greater reductions in LDL-C levels compared with statins alone. This enhanced LDL particle reduction largely mediates decreases in other apolipoproteins, including APOC2, APOC3, and APOE, as shown for the first time by mediation analysis. Notably, a post-MI rise of Lp(a) was observed in the placebo group but not in patients treated with PCSK9 mAbs, supporting their use to attenuate Lp(a) increases in patients initiating high-intensity statins after MI until specific Lp(a)-lowering therapies become clinically available. With the growing recognition of apolipoproteins in risk assessment and as therapeutic targets, future research should extend beyond APOB and APOA1 to refine risk stratification and treatment response in secondary prevention.

## ARTICLE INFORMATION

### Acknowledgments

This research has been conducted using the UK Biobank Resource under application number 22102.

### Sources of Funding

Dr Mayr is a British Heart Foundation (BHF) Chair Holder with BHF program and project grant support (CH/16/3/32406, RG/F/21/110053, PG/20/10387). Dr Mayr is also supported by the Leducq Foundation (18CVD02) and the excellence initiative VASCage (Centre for Promoting Vascular Health in the Ageing Community, project number 868624) of the Austrian Research Promotion Agency FFG (COMET program–Competence Centers for Excellent Technologies) funded by the Austrian Ministry for Transport, Innovation and Technology; the Austrian Ministry for Digital and Economic Affairs; and the federal states Tyrol (via Standortagentur), Salzburg, and Vienna (via Vienna Business Agency). Dr Theofilatos is supported by BHF Centre Pump Prime Award RE25626. This work is supported by Imperial BHF Research Excellence Award (4) - RE/24/130023.

### Disclosures

Dr Lang received travel support from Medtronic during the conduct of the study and grants and personal fees from Janssen, AOP Orphan, MSD, and Neutrolis outside the submitted work. Dr Koskinas received grants from Sanofi, Regeneron, and Infraredx during the conduct of the study and personal fees from Amgen and Daiichi Sankyo outside the submitted work. Dr Speidl received speaker fees from Daiichi Sankyo, Amgen, and Amarin; served as a consultant to Daiichi Sankyo, Amgen, Amarin, and Sanofi; and received travel support from Amgen. Dr Räber received grants from Sanofi, Regeneron, and Infraredx to Inselspital and speaker fees from Sanofi during the conduct of the study; and received grants from Abbott, Heartflow, Boston Scientific, and Biotronik to Inselspital, as well as speaker and consultation fees from Abbott, Amgen, AstraZeneca, Gentuity, Occlutech, Sanofi, Canon, and Medtronic outside the submitted work. The other authors report no conflicts.

### Supplemental Material

Supplemental Methods

Tables S1–S9

Figures S1–S11

References 48–58

## Supplementary Material
