# Supplementary material for: Integrative Proteomic and Lipidomic Analysis of Patients With Acute Myocardial Infarction Treated With PCSK9 Antibodies and Statins
Source: Circ Genom Precis Med. 2026 Feb 10;19(2):e005345. doi: 10.1161/CIRCGEN.125.005345 (PMC13102353; doi:10.1161/CIRCGEN.125.005345)
Supplement: Supplementary file 1 [file hcg-19-e005345-s001.pdf]

SUPPLEMENTAL MATERIAL

Integrative Proteomic and Lipidomic Analysis of Patients With Acute Myocardial Infarction Treated With PCSK9 Antibodies and Statins

Lukas E. Schmidt, MSc[1], Sean A. Burnap, PhD[2,3], Bhawana Singh, PhD[4], Kaloyan Takov, PhD[4], Sylvain Losdat, PhD[5], Lore Schrutka, MD, PhD[1], Lukas Galli, MD[1], Konstantinos Theofilatos, PhD[6], Georg W. Otto, PhD[7], Christian Hengstenberg, MD[1], Ioanna Tzoulaki, PhD[7], Irene M. Lang, MD[1], Konstantinos C. Koskinas, MD, MSc[8], Walter S. Speidl, MD[1], Lorenz Räber, MD, PhD[8], Manuel Mayr, MD, PhD[1,4]

[1] Division of Cardiology, Department of Internal Medicine II, Medical University of Vienna, Austria
[2] Department of Biochemistry, University of Oxford, United Kingdom
[3] The Kavli Institute for Nanoscience Discovery, University of Oxford, United Kingdom
[4] National Heart and Lung Institute, Imperial College London, United Kingdom
[5] Department of Clinical Research, University of Bern, Switzerland
[6] British Heart Foundation Centre of Research Excellence, School of Cardiovascular and Metabolic Medicine & Sciences, King's College London, United Kingdom
[7] Department of Epidemiology and Biostatistics, School of Public Health, Imperial College London, United Kingdom
[8] Department of Cardiology, University of Bern, Switzerland

Address for correspondence: Prof. Manuel Mayr, MD, PhD, Sir Michael Uren Hub, White City Campus, Imperial College London, 86 Wood Lane, London W12 0BZ, United Kingdom. Telephone: +44 20 75949909. E-mail: m.mayr@imperial.ac.uk.

SUPPLEMENTAL METHODS

PACMAN-AMI trial

The PACMAN-AMI (Effects of the PCSK9 Antibody Alirocumab on Coronary Atherosclerosis in Patients With Acute Myocardial Infarction) trial was an investigator-initiated, multicenter, double-blind, placebo-controlled, randomized trial which evaluated the effect of alirocumab on coronary atherosclerosis by multi-modality intracoronary imaging in patients presenting with acute MI who underwent urgent percutaneous coronary intervention (PCI). Details about the trial design, patient population, trial procedures, and endpoints have been published previously[9,48]. 300 patients were randomized to receive either 150 mg biweekly alirocumab (N = 148) or placebo (N = 152). Additionally, all patients in both treatment groups were started on a high-intensity statin (20 mg rosuvastatin daily) following MI, and adherence was confirmed by direct measurement of statin blood concentrations at four and 52 weeks[49]. Blood samples were immediately processed and stored at -80 °C locally, and subsequently transferred to a central biobank. Longitudinal plasma samples of 265 acute MI patients who completed the 52-week follow-up were available for analysis. The PACMAN-AMI trial was approved by all local ethics authorities of the participating centers, with Kantonale Ethikkommission Bern, Switzerland, acting as the lead competent ethics committee. The pre-registered clinical trial number is NCT03067844 (registry: ClinicalTrials.gov).

Vienna Lipid Clinic prospective registry

The Vienna Lipid Clinic prospective registry included hyperlipidemic patients receiving PCSK9 mAb treatment. The study cohort was followed up for six months, with blood samples taken at baseline, after four weeks, and after six months. Blood samples were immediately processed and stored at -80 °C at a local biobank. Longitudinal plasma samples of 34 patients were available for analysis. The Vienna Lipid Clinic prospective registry was established in

accordance with the Declaration of Helsinki and approved by the Ethics Committee of the Medical University of Vienna (EK no. 1706/2018). Written informed consent was obtained from all participants prior to their inclusion in the study.

Discovery proteomics by data-independent acquisition–mass spectrometry (DIA–MS)

The DIA–MS analysis of EDTA plasma from 44 patients in the PACMAN-AMI Bern subcohort (**Table S2**) was blinded. Plasma proteins were denatured, reduced, alkylated and subjected to an in-solution digestion with trypsin/Lys-C (Promega). Acidified peptides were desalted using C18 cartridges on an AssayMAP Bravo Liquid Handling Platform (Agilent). Eluted peptides were vacuum centrifuged (Thermo Scientific, Savant SPD131DDA) and resuspended in 2% acetonitrile (ACN), 0.05% TFA (aq).

The DIA–MS experiment was conducted on an UltiMate 3000 high-performance liquid chromatography (HPLC) system equipped with a capillary flow selector, which was coupled via an EASY-Spray NG source to an Orbitrap Fusion Lumos mass spectrometer (all Thermo Scientific). Peptides were loaded directly onto an analytical column (Thermo Scientific, EASY-Spray, 150 µm inner diameter × 15 cm length, 2 µm particle size, 100 Å pore size) kept at 50 °C. Loading was performed using 0.1% formic acid (FA, aq) at a flow rate of 2.5 µL/min. Peptide separation was achieved at 1.2 µL/min with the following gradient: 3–5 min, 6–9% B; 5–11 min, 9–14% B; 11–17 min, 14–19% B; 17–23 min, 19–24% B; 23–30 min, 24–30% B; 30–35 min, 30–51% B. A wash step at 99% B for 5 minutes was followed by equilibration at 4% B for 5 minutes, both at a flow rate of 2.5 µL/min. Mobile phase A was 0.1% FA (aq) and mobile phase B was 0.1% FA in 80% ACN (aq). Precursor ions were analyzed in the Orbitrap (resolution 60,000, scan range 330–1,201 m/z). DIA of fragment spectra (25 variable m/z windows) was performed after higher-energy collisional dissociation (HCD) and Orbitrap analysis (resolution 30,000).

DIA–MS data were analyzed in Spectronaut (Biognosys, v14) using a library-based search against an in-house spectral library (8,628 precursors) generated from 22 fractions of a plasma pool depleted with Multiple Affinity Removal System spin columns (Agilent). The Q value cut-off was set to 0.01. Quantification was based on MS2 peak areas. The global normalization algorithm in Spectronaut was set to median and used for normalization. Proteins with more than 30% missing values were filtered out and the remaining missing values were imputed by multiplying the lowest quantified value per protein by 0.5.

UK Biobank (UKB) clinical APOA1, APOB, and Lp(a) assay data

Plasma proteomic data, measured using the antibody-based Olink Explore 3072 platform, are available for a subcohort of 54,219 UKB participants[10]. Clinical APOA1, APOB, and Lp(a) assays were used as orthogonal validation of the corresponding Olink assays. APOA1 was measured at baseline in the serum of 427,211 UKB participants using immunoturbidimetric analysis on a Beckman Coulter AU5800 (UKB data-field 30630). APOB was measured at baseline in the serum of 466,883 UKB participants using clinical assay immunoturbidimetric analysis on a Beckman Coulter AU5800 (UKB data-field 30640). Lp(a) was measured in the serum of 375,367 UKB participants using immunoturbidimetric analysis on a Randox AU5800 (UKB data-field 30790). After excluding participants with missing APOA1, APOB, or Lp(a) measurements, as well as those who had withdrawn from the study, 336,173 individuals with complete clinical assay data were retained for analysis.

Atorvastatin-treated HepG2 human hepatoma cells stably expressing LPA

HepG2 human hepatoma cells (ECACC, 85011430) were maintained in Dulbecco's Modified Eagle Medium supplemented with 10% fetal bovine serum (FBS, Merck), 2 mM L-alanyl-L-glutamine (Gibco, Glutamax), 100 U/ml penicillin and 100 µg/ml streptomycin. A plasmid encoding LPA (Origene, RC212070) was used to generate HepG2 cells stably expressing LPA.

DDK and myc tags on LPA were replaced with a HiBiT tag using NEBuilder HiFi DNA Assembly (NEB) and standard cloning procedures. The HiBiT tag allows for easy detection of secreted LPA by a split luciferase assay[50] and therefore quantifiable measurement of Lp(a) release from the cells. HepG2 cells were transfected with the modified vector using Viafect (Promega) according to the manufacturer's instructions. The plasmid encodes resistance to neomycin. 48 h after transfection, cells were treated with Geneticin (Gibco) at 1,600 µg/ml in the medium used for culturing (see above) devoid of penicillin and streptomycin. Cells were maintained in these conditions for at least a month to allow for selection of cells that stably produce Lp(a). Geneticin concentration was then reduced to 800 µg/ml for maintenance of the polyclonal populations of cells. For atorvastatin treatment, LPA-expressing HepG2 cells were cultured in complete medium containing 0.1, 1, or 10 µM atorvastatin for nine days, followed by incubation in serum-free medium with the same atorvastatin concentration for the final 24 h. Vehicle was dimethyl sulfoxide. Conditioned media and cell lysates were collected for further analyses.

Immunoblotting

For Immunoblotting, Laemmli buffer and 2-mercaptoethanol were added to the samples and mixtures were boiled for 10 min at 80 °C. Gel electrophoresis was performed using either 4–12% Bis-Tris or 3–8% Tris-Acetate gradient gels (Invitrogen). Where indicated, gels were stained for total protein using SimplyBlue Safe Stain (Invitrogen) or proteins were transferred onto nitrocellulose membranes. Ponceau S staining was then performed before membranes were blocked in 5% fat-free milk in phosphate-buffered saline containing 0.1% Tween-20 (PBS-T, Sigma-Aldrich). Membranes were incubated in primary antibodies in 5% (w/v) bovine serum albumin in PBS-T overnight at 4 °C or for 2 h at room temperature. This was followed by incubation of the membranes with IRDye fluorescent dye-secondary antibody conjugates (LICOR) in 5% milk/PBS-T. An Odyssey CLx scanner (LICOR) was used for fluorescence

detection. Primary antibodies included anti-APOA1 (Academy Bio-Medical), anti-APOB (Invitrogen, clone F2C9), anti-LPA (Sigma-Aldrich, clone LPA4), and anti-PCOLCE (Abcam, ab39204).

Discovery proteomics by data-dependent acquisition–mass spectrometry (DDA–MS)

Serum-free medium conditioned by HepG2 cells was cleared of debris by centrifugation and concentrated by ultrafiltration (Merck, Amicon regenerated cellulose membrane). In-solution digestion of conditioned media and cell lysates was performed as described above. The DDA–MS experiments were conducted on a Vanquish Neo ultra high-performance liquid chromatography (UHPLC) system that was coupled via an EASY-Spray source to an Orbitrap Eclipse mass spectrometer equipped with a high-field asymmetric waveform ion mobility spectrometry (FAIMS) Pro interface (all Thermo Scientific). A 35-minute gradient with increasing strength of mobile phase B [80% ACN, 0.1% FA (aq)] was used to elute the peptides from a trap cartridge (Thermo Scientific, PepMap Neo, 300 µm inner diameter × 5 mm length, 5 µm particle size, 100 Å pore size) at 0.25 µL/min. The peptides were separated on a reversed-phase analytical column (Thermo Scientific, EASY-Spray PepMap Neo, 75 µm inner diameter × 50 cm length, 2 µm particle size, 100 Å pore size) kept at 60 °C. Loading and washing were performed at 0.45 µL/min (total run time 50 min). FAIMS compensation voltages of -40 V, -60 V, and -80 V were applied. Precursor ions were analyzed in the Orbitrap (resolution 120,000, scan range 375–1,500 m/z). Data-dependent fragment spectra were acquired after HCD and ion trap analysis (Cycle Time mode) using a fixed total cycle time of 3 s and a dynamic exclusion duration of 60 s.

DDA–MS raw files were processed using Proteome Discoverer (Thermo Scientific, v3.2.0.450) and Mascot (Matrix Science, v2.999.0). The files were searched against a human database (UniProtKB/Swiss-Prot, release 2025_01, 20,417 proteins) and in-house databases compiled from mass spectrometric analyses of FBS (249 bovine proteins, porcine trypsin, and

Lys-C from *P. aeruginosa*) and tagged human apolipoproteins (23 proteins). Search results were filtered to include human master proteins with a high protein FDR confidence, as determined by Proteome Discoverer's FDR Validator node (FDR confidence threshold of 0.01 for high confidence), and a minimum number of two unique peptides. Only proteins with <20% missing values in at least one atorvastatin dose groups were retained. Missing values were KNN-imputed (K = 5) using the *DMwR2* package (v0.0.2) when ≤30% of values were missing across all samples. When >30% of values were missing across all samples but <20% were missing in at least one group, and >50% were missing in the remaining groups, the missing values were imputed by multiplying the lowest quantified value per protein by 0.2.

Targeted apolipoprotein proteomics by parallel reaction monitoring–mass spectrometry (PRM–MS)

Apolipoproteins were measured by PRM–MS. The PRM–MS analyses of EDTA plasma were blinded. Plasma proteins were denatured and reduced by incubation with 7.2 M urea and 5 mM dithiothreitol at 37 °C with 180 rpm orbital shaking for 1 h. The reduced proteins were cooled to room temperature and alkylated in the dark with 25 mM iodoacetamide for 30 min. Alkylated proteins were diluted with 0.1 M triethylammonium bicarbonate (pH 8.5) containing a mix of stable isotope-labeled standard (SIS) peptides (Thermo Scientific, HeavyPeptide AQUA Ultimate; **Table S9**). Proteins were digested with 0.4 µg of Trypsin/Lys-C at 37 °C with 180 rpm orbital shaking for 18 h. The resulting peptide solutions were acidified to a final concentration of 1% TFA. Peptide clean-up was performed using C18 cartridges on an AssayMAP Bravo Liquid Handling Platform (Agilent). Eluted peptides were vacuum centrifuged (Thermo Scientific, Savant SPD131DDA) and resuspended in 2% ACN, 0.05% TFA (aq).

The PRM–MS experiments were conducted on a Vanquish UHPLC system coupled via an Ion Max or Ion Max NG electrospray ion source with a HESI-II probe to a Q Exactive HF

or Orbitrap Fusion Lumos mass spectrometer (all Thermo Scientific) for the acute MI and non-MI cohorts, respectively. Peptides were separated on an AdvanceBio Peptide Mapping column (Agilent, 2.1 mm inner diameter × 25 cm length, 2.7 µm particle size, 120 Å pore size, kept at 50 °C) at a flow rate of 0.35 mL/min. The following gradient was used: 0–0.1 min, 5–10% B; 0.1–9 min, 10–32.5% B; 9–10 min, 32.5–80% B; 10–12 min, 80% B; 12–12.2 min, 80–5% B; 12.2–15 min, 5% B. Mobile phase A was 0.1% FA (aq), and mobile phase B was 0.1% FA in ACN. Targeted peptides were analyzed by PRM using HCD fragmentation and detection of fragments in the Orbitrap (resolution 15,000).

PRM–MS data were analyzed in SpectroDive (Biognosys, v10). Quantification was based on the peak area sum of all selected transitions. The known amount of SIS peptides facilitated the calculation of light-to-heavy ratios and absolute molar concentrations for each peptide. Where applicable, peptide molar concentrations were converted to protein mass concentrations using the protein's molecular weight. Peptide molar concentrations and protein mass concentrations below the limit of quantification were imputed by multiplying the lowest quantified value per peptide or protein by 0.5.

Targeted lipidomics by flow injection analysis–mass spectrometry (FIA–MS)

Lipids were extracted from EDTA plasma from 35 patients in the PACMAN-AMI Bern subcohort (**Table S8**) and analyzed using the AbsoluteIDQ p400 HR kit (Biocrates Life Sciences) according to the manufacturer's instructions. Lipid extracts were analyzed by FIA–MS on a Vanquish UHPLC system coupled via an Ion Max electrospray ion source with a HESI-II probe to a Q Exactive HF mass spectrometer (all Thermo Scientific). Raw data were processed using the supplied MetIDQ software (Biocrates Life Sciences, v6.4.8). Only lipids that had a concentration greater than that of the limit of quantification were taken forward for analysis. Lipids with more than 30% missing values were filtered out and the remaining missing values were imputed by multiplying the lowest quantified value per lipid by 0.5.

Statistical analysis of MS data

In-house MS-based proteomic and lipidomic data were analyzed using R (The R Foundation for Statistical Computing, v4.3.3). Comparisons of continuous clinical variables were performed using the unpaired Wilcoxon rank-sum test, while the Chi-squared test was used for binary variables. Quantitative values were analyzed after $\log_2$ transformation. Missing values in proteomic and lipidomic datasets were accepted up to 30% and imputed as outlined above. Differential expression analysis of DIA–MS data was performed using the *limma* package (v3.58.1). Multiple testing correction was applied using the Benjamini-Hochberg method to control the false discovery rate. For conditioned media DDA–MS data, two-way repeated-measures analysis of variance was used to assess the effect of atorvastatin treatment. Correlations between apolipoprotein levels assessed by MS and clinically determined lipid levels were evaluated using Spearman correlation. Longitudinal analyses of apolipoprotein and clinically determined lipid data were performed using linear mixed-effects models (*lme4* package, v1.1.35.1) to assess the impacts of timepoint and treatment variables. The Wilcoxon rank-sum test was used for independent comparisons between treatments, while the Wilcoxon signed-rank test was applied for paired comparisons between two specific timepoints. For visualization of effect sizes in forest plots, four-week changes from baseline or from the 24-hour timepoint were transformed from a logarithmic scale to a percentage scale.

R packages

The following R packages were used: *DMwR2* (v0.0.2)[51], *ggplot2* (v3.5.0)[52], *impute* (v1.80.0)[53], *limma* (v3.58.1)[54], *lme4* (v1.1.35.1)[55], *mediation* (v4.5.0)[56], and *patchwork* (v1.2.0)[57].

Data availability

Mass spectrometry proteomics data from the hepatoma cell experiments have been deposited to the ProteomeXchange Consortium via the PRIDE[58] partner repository with the digital object identifier 10.6019/PXD069776.

SUPPLEMENTAL TABLES

Table S1. Baseline characteristics of 85 acute MI patients from the PACMAN-AMI Bern subcohort, from whom additional blood was collected 24 hours after PCI.

Variable	PCSK9 mAb (N = 38)	Placebo (N = 47)	P value
Age (years)	**59** (53, 65)	**59** (53, 65)	0.87
Female sex (%)	**13.2**	**21.3**	0.49
BMI (kg/m^2)	**25.4** (23.8, 29.0)	**28.0** (26.1, 31.2)	0.04
Systolic blood pressure (mm Hg)	**132** (115, 144)	**127** (122, 141)	0.57
Diastolic blood pressure (mm Hg)	**76** (69, 88)	**71** (66, 81)	0.11
Family history of CAD (%)	**26.3**	**36.2**	0.46
Diabetes (%)	**5.3**	**12.8**	0.42
Current smoker (%)	**42.1**	**27.7**	0.24
History of smoking (%)	**60.5**	**61.7**	1.00
Statin use (%)	**13.2**	**17.0**	0.85
Total cholesterol (mmol/L)	**5.2** (4.9, 5.7)	**5.4** (5.0, 6.2)	0.39
HDL-C (mmol/L)	**1.1** (0.9, 1.3)	**1.1** (0.9, 1.3)	0.87
LDL-C (mmol/L)	**4.0** (3.6, 4.4)	**4.0** (3.6, 4.8)	0.69
Triglycerides (mmol/L)	**0.9** (0.6, 1.4)	**1.0** (0.7, 1.3)	0.31
Lipoprotein(a) (nmol/L)	**29** (9, 97)	**20** (9, 135)	0.86

Values are median (25th, 75th percentile) for continuous variables. Continuous variables were analyzed with the Wilcoxon rank-sum test, binary variables with the Chi-squared test. Statistical tests are two-sided and unadjusted. BMI, body mass index; CAD, coronary artery disease; HDL-C, high-density lipoprotein-cholesterol; LDL-C, low-density lipoprotein-cholesterol; mAb, monoclonal antibody; MI, myocardial infarction; PCI, percutaneous coronary intervention; PCSK9, proprotein convertase subtilisin/kexin type 9.

Table S2. Baseline characteristics of 44 acute MI patients from the PACMAN-AMI Bern subcohort used for discovery proteomics.

Variable	PCSK9 mAb (N = 21)	Placebo (N = 23)	P value
Age (years)	**54** (51, 58)	**59** (53, 63)	0.10
Female sex (%)	**0**	**26.1**	0.04
BMI (kg/m^2)	**25.0** (23.9, 30.1)	**28.0** (25.3, 30.1)	0.26
Systolic blood pressure (mm Hg)	**132** (117, 145)	**127** (117, 137)	0.30
Diastolic blood pressure (mm Hg)	**84** (73, 91)	**72** (67, 81)	0.04
Family history of CAD (%)	**33.3**	**34.8**	1.00
Diabetes (%)	**4.8**	**13.0**	0.67
Current smoker (%)	**47.6**	**30.4**	0.39
History of smoking (%)	**66.7**	**65.2**	1.00
Statin use (%)	**19.0**	**21.7**	1.00
Total cholesterol (mmol/L)	**5.2** (5.1, 5.6)	**5.4** (5.1, 6.3)	0.29
HDL-C (mmol/L)	**1.0** (0.9, 1.2)	**1.1** (1.0, 1.4)	0.17
LDL-C (mmol/L)	**4.0** (3.8, 4.3)	**3.9** (3.8, 5.0)	0.65
Triglycerides (mmol/L)	**0.9** (0.8, 1.6)	**1.0** (0.7, 1.3)	0.67
Lipoprotein(a) (nmol/L)	**30** (9, 98)	**45** (8, 127)	0.92

Data relates to discovery proteomics data shown in Figure 1A. Values are median (25th, 75th percentile) for continuous variables. Continuous variables were analyzed with the Wilcoxon rank-sum test, binary variables with the Chi-squared test. Statistical tests are two-sided and unadjusted. BMI, body mass index; CAD, coronary artery disease; HDL-C, high-density lipoprotein-cholesterol; LDL-C, low-density lipoprotein-cholesterol; mAb, monoclonal antibody; MI, myocardial infarction; PCI, percutaneous coronary intervention; PCSK9, proprotein convertase subtilisin/kexin type 9.

Table S3. Demographic characteristics of UK Biobank participants used in the comparison of statin users and non-users, including participants receiving any other medications.

Variable	Statin (N = 4,817)	No statin (N = 25,815)	P value
Age (years)	**63** (58, 66)	**57** (49, 63)	$< 2.2 \times 10^{-16}$
Female sex (%)	**37**	**57**	$< 2.2 \times 10^{-16}$

Data relates to UK Biobank protein data shown in Figures S1 and S3. Age is presented as median (25th, 75th percentile). P values (statin versus no statin) were computed using the Wilcoxon rank-sum test for age and Fisher's exact test for sex. Statistical tests were two-sided and unadjusted.

Table S4. Demographic characteristics of subset of UK Biobank participants used in the comparison of statin users and non-users, excluding participants receiving any other medications.

Variable	Statin (N = 357)	No statin (N = 11,657)	P value
Age (years)	**61** (56, 65)	**54** (47, 61)	$< 2.2 \times 10^{-16}$
Female sex (%)	**35**	**48**	2.6×10^{-6}

Data relates to UK Biobank protein data shown in Figure S2. Age is presented as median (25th, 75th percentile). P values (statin versus no statin) were computed using the Wilcoxon rank-sum test for age and Fisher's exact test for sex. Statistical tests were two-sided and unadjusted.

Table S5. Demographic characteristics of subset of UK Biobank participants with detectable PCOLCE levels used in the comparison of statin users and non-users, excluding participants receiving any other medications.

Variable	Statin (N = 89)	No statin (N = 3,035)	P value
Age (years)	**62** (58, 65)	**55** (47, 61)	1.3×10^{-13}
Female sex (%)	**40**	**48**	0.197

Data relates to UK Biobank protein data shown in Figures 1B and C. Age is presented as median (25th, 75th percentile). P values (statin versus no statin) were computed using the Wilcoxon rank-sum test for age and Fisher's exact test for sex. Statistical tests were two-sided and unadjusted. PCOLCE, procollagen C-endopeptidase enhancer 1.

Table S6. Mediation analysis including covariates age, sex, baseline statin status, and baseline LDL-C.

	Direct Treatment Effect			Indirect Effect via LDL-C		
	Log_2 ADE	ADE (%)	Adj. P value	Log_2 ACME	ACME (%)	Adj. P value
Lp(a)	-0.27	-17.0	0.11	-0.24	-15.6	0.04
LPA	-0.12	-8.2	0.74	-0.68	-37.5	0.05
APOB	-0.14	-9.3	0.11	-1.02	-50.8	<0.001
Triglycerides	0.08	5.4	0.68	-0.40	-24.0	<0.001
APOC1	-0.28	-17.7	0.43	-0.20	-12.7	0.50
APOC2	0.28	21.5	0.27	-0.58	-33.2	<0.001
APOC3	0.21	15.9	0.19	-0.45	-26.7	<0.001
APOE	0.07	5.0	0.74	-0.72	-39.3	<0.001
APOD	0.18	12.9	0.27	-0.39	-23.9	<0.001
APOL1	0.19	14.4	0.21	-0.36	-22.1	<0.001
APOM	0.23	17.3	0.11	-0.41	-24.9	<0.001

Summary of direct and indirect effects from the mediation analysis visualized in Figure S8. Mediator and outcome models included covariates age, sex, baseline statin status, and baseline LDL-C. Only parameters with significantly greater reductions in the PACMAN-AMI alirocumab arm compared to placebo (adj. $P < 0.05$, Figure 3A) were included. P values were adjusted for multiple testing using the Benjamini-Hochberg method. Note that percent changes estimated by mediation models are based on regression outputs and may differ from median percent changes shown in Figure 3A. ACME, average causal mediation effect; ADE, average direct effect; adj., adjusted; APO, apolipoprotein; LDL-C, low-density lipoprotein cholesterol; LPA, apolipoprotein(a); Lp(a), lipoprotein(a); PACMAN-AMI, Effects of the PCSK9 Antibody Alirocumab on Coronary Atherosclerosis in Patients With Acute Myocardial Infarction; PCSK9, proprotein convertase subtilisin/kexin type 9.

Table S7. Baseline characteristics of 34 non-MI patients from the Vienna Lipid Clinic cohort who received PCSK9 mAb therapy compared with 131 acute MI patients from the PACMAN-AMI cohort (PCSK9 mAb group).

Variable	Non-MI (N = 34)	Acute MI (N = 131)	P value
Age (years)	**58** (51, 65)	**58** (51, 65)	0.86
Female sex (%)	**47.1**	**14.5**	9.5×10^{-5}
BMI (kg/m^2)	**27.5** (25.1, 29.2)	**27.2** (24.5, 30.4)	0.82
CHD (%)	**88.2**	NA	NA
Diabetes (%)	**20.6**	**8.4**	0.085
Current or former smoker (%)	**58.8**	**68.7**	0.38
Statin use (%)	**73.5**	**11.5**	2.8×10^{-13}
Total cholesterol (mmol/L)	**4.6** (3.6, 5.1)	**5.2** (4.7, 6.0)	7.2×10^{-5}
HDL-C (mmol/L)	**1.2** (1.1, 1.4)	**1.0** (0.9, 1.2)	2.8×10^{-4}
LDL-C (mmol/L)	**2.4** (1.8, 3.1)	**4.0** (3.5, 4.5)	2.4×10^{-11}
Triglycerides (mmol/L)	**1.3** (0.9, 1.8)	**1.0** (0.7, 1.5)	0.015
Lipoprotein(a) (nmol/L)	**139** (8, 236)	**15** (7, 86)	0.0054

Values are median (25th, 75th percentile) for continuous variables. BMI, body mass index; CHD, coronary heart disease; HDL-C, high-density lipoprotein-cholesterol; LDL-C, low-density lipoprotein-cholesterol; mAb, monoclonal antibody; MI, myocardial infarction; PCSK9, proprotein convertase subtilisin/kexin type 9.

Table S8. Baseline characteristics of 35 acute MI patients from the PACMAN-AMI Bern subcohort used for targeted lipidomics.

Variable	PCSK9 mAb (N = 20)	Placebo (N = 15)	P value
Age (years)	**55** (52, 60)	**61** (54, 65)	0.13
Female sex (%)	**0**	**26.7**	0.06
BMI (kg/m^2)	**25.1** (23.7, 30.7)	**27.8** (24.9, 29.1)	0.78
Systolic blood pressure (mm Hg)	**131** (116, 136)	**123** (111, 130)	0.32
Diastolic blood pressure (mm Hg)	**83** (70, 90)	**71** (62, 73)	0.01
Family history of CAD (%)	**35.0**	**33.3**	1.00
Diabetes (%)	**5.0**	**6.7**	1.00
Current smoker (%)	**40.0**	**40.0**	1.00
History of smoking (%)	**60.0**	**66.7**	0.96
Statin use (%)	**20.0**	**13.3**	0.95
Total cholesterol (mmol/L)	**5.3** (5.1, 5.8)	**5.7** (5.2, 6.4)	0.23
HDL-C (mmol/L)	**1.0** (0.9, 1.2)	**1.1** (1.0, 1.3)	0.22
LDL-C (mmol/L)	**4.1** (3.7, 4.4)	**4.0** (3.8, 5.1)	0.66
Triglycerides (mmol/L)	**0.9** (0.7, 1.7)	**1.2** (0.7, 1.5)	0.85
Lipoprotein(a) (nmol/L)	**33** (9, 100)	**13** (9, 89)	0.81

Data relates to targeted lipidomics data shown in Figures 5 and 6. Values are median (25th, 75th percentile) for continuous variables. Continuous variables were analyzed with the Wilcoxon rank-sum test, binary variables with the Chi-squared test. Statistical tests are two-sided and unadjusted. BMI, body mass index; CAD, coronary artery disease; HDL-C, high-density lipoprotein-cholesterol; LDL-C, low-density lipoprotein-cholesterol; mAb, monoclonal antibody; MI, myocardial infarction; PCI, percutaneous coronary intervention; PCSK9, proprotein convertase subtilisin/kexin type 9.

Table S9. Stable isotope-labeled standards.

Protein	AQUA Ultimate peptide sequence	Concentration acute MI cohort (nM)	Concentration non-MI cohort (nM)
APOA1	VSFLSALEEYTK[+8]	357	200
APOA2	EQLTPLIK[+8]	238	150
APOA4	LGEVNTYAGDLQK[+8]	42	25
APOB	TEVIPPLIENR[+10]	30	50
APOC1	EFGNTLEDK[+8]	30	50
APOC2	TAAQNLYEK[+8]	30	50
APOC3	GWVTDGFSSLK[+8]	179	100
APOD	NILTSNNIDVK[+8]	30	25
APOE	SELEEQLTPVAEETR[+10]	12	25
APOH	ATVVYQGER[+10]	60	50
APOL1	ALDNLAR[+10]	12	25
APOM	FLLYNR[+10]	18	25
APOJ	ASSIIDELFQDR[+10]	119	50
LPA	GTYSTTVTGR[+10]	12	25

AQUA Ultimate peptides were obtained from Thermo Scientific. Arginine and lysine residues of the peptides were labeled with ^{13}C and ^{15}N atoms. Values in square brackets refer to the approximate mass shift in Dalton of a given stable isotope-labeled peptide compared to the unlabeled peptide. The LPA peptide is part of the kringle IV type 2 repeat region. APO, apolipoprotein; LPA, apolipoprotein(a); MI, myocardial infarction.

SUPPLEMENTAL FIGURES

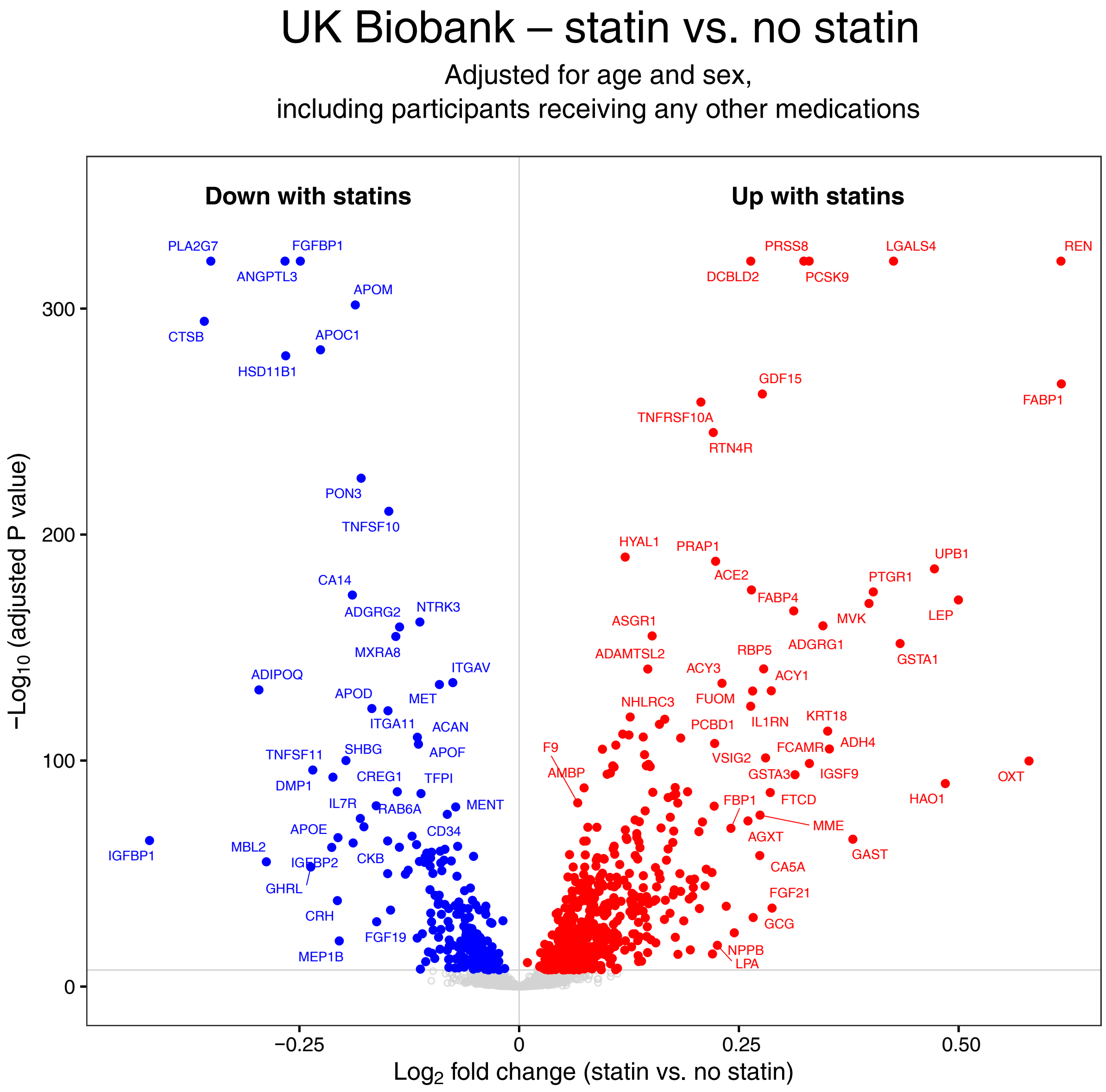

Figure S1. Association between plasma protein levels and statin use in UK Biobank participants.
Differences in plasma protein levels between statin users (N = 4,817) and statin-naïve participants (N = 25,815), including participants receiving any other medications besides statins. Plasma protein levels of UK Biobank participants were quantified using the Olink Explore 3072 platform. Significance threshold was set at adjusted P = 5×10^{-8}. Adjusted P values for ANGPTL3, DCBLD2, FGFBP1, LGALS4, PCSK9, PLA2G7, PRSS8, and REN were returned as zero due to high statistical significance and, for visualisation purposes, were set to 1×10^{-321}. P values were adjusted for multiple testing using the Benjamini-Hochberg method.

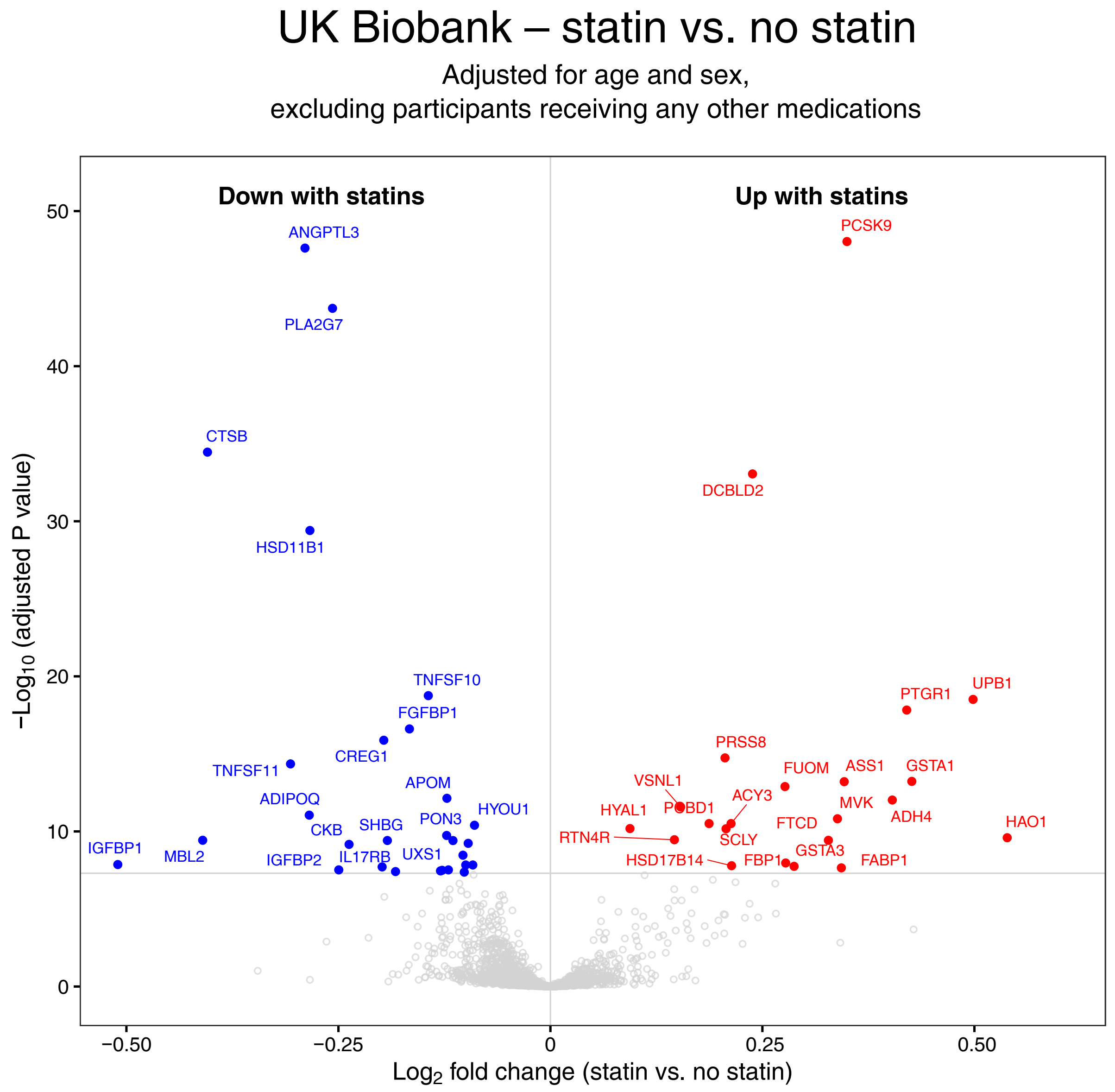

Figure S2. Association between plasma protein levels and statin use in UK Biobank participants.
Differences in plasma protein levels between statin users (N = 357) and statin-naïve participants (N = 11,657), excluding participants receiving any other medications besides statins. Plasma protein levels of UK Biobank participants were quantified using the Olink Explore 3072 platform. Significance threshold was set at adjusted P = 5 × 10^{-8}. P values were adjusted for multiple testing using the Benjamini-Hochberg method.

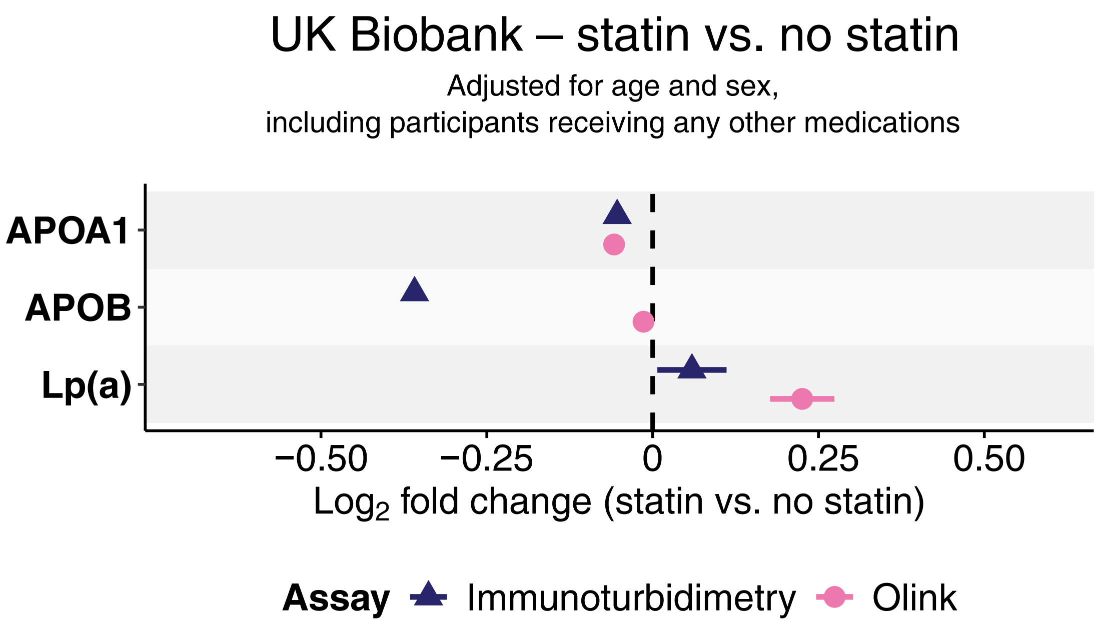

Figure S3. Comparison of immunoturbidimetric and Olink assays for APOA1, APOB, and Lp(a) in UK Biobank participants.
Comparison between statin users (N = 4,817) and statin-naïve participants (N = 25,815), including participants receiving any other medications besides statins. Error bars indicate the 95% confidence interval. Olink values are reported as the LPA protein, while the immunoturbidimetric assay measures the Lp(a) particle. APO, apolipoprotein; LPA, apolipoprotein(a); Lp(a), lipoprotein(a).

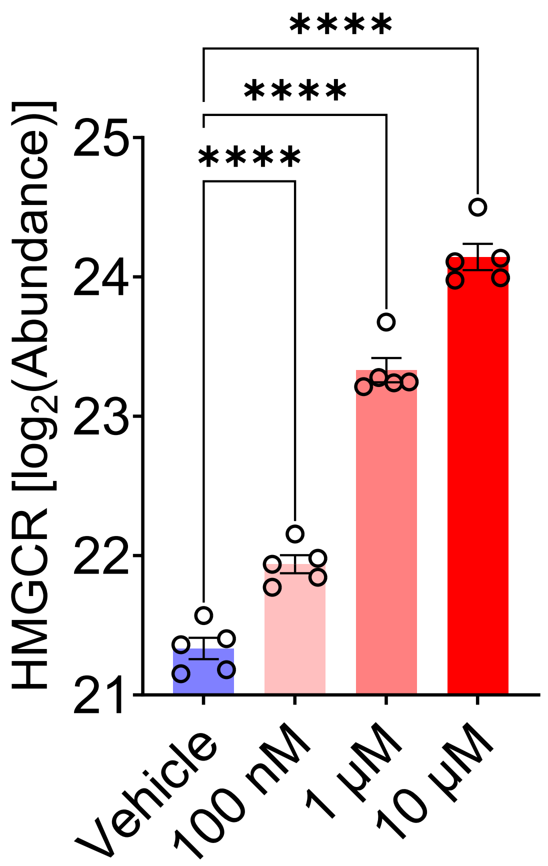

Figure S4. HMGCR quantification in lysates of atorvastatin-treated HepG2 cells. Medium of HepG2 cells expressing LPA was conditioned with the indicated concentrations of atorvastatin for ten days. Quantification of cell lysates was done by mass spectrometry. Significant P values from two-way repeated-measures analysis of variance are indicated by asterisks: ****, P value < 0.0001. Vehicle was dimethyl sulfoxide. HMGCR, HMG-CoA reductase; LPA, apolipoprotein(a).

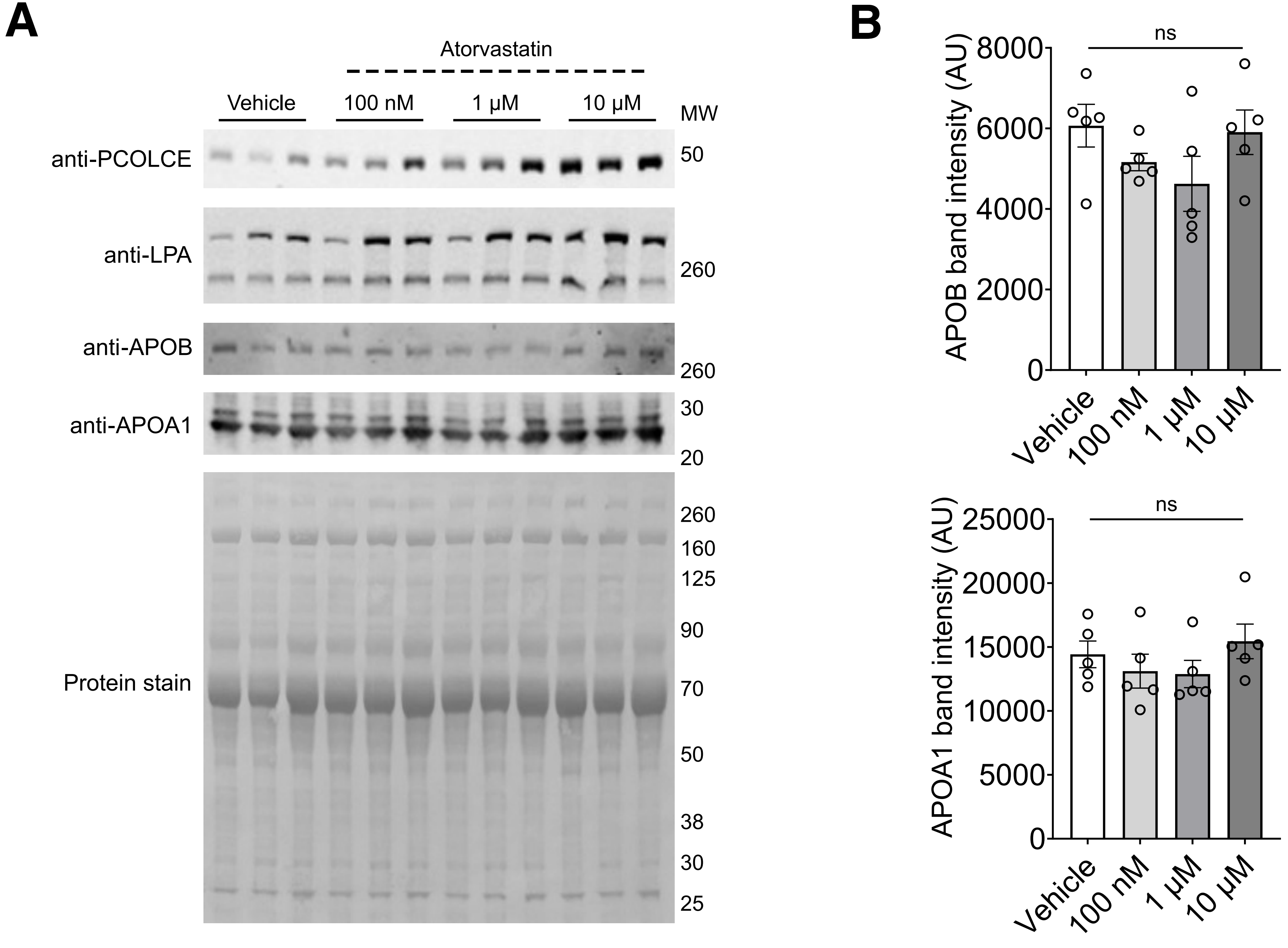

Figure S5. Protein responses in conditioned media of atorvastatin-treated HepG2 cells.
Medium of HepG2 cells expressing LPA was conditioned with the indicated concentrations of atorvastatin for ten days. Immunoblots for indicated proteins (**A**) and associated quantification by densitometry (**B**). APO, apolipoprotein; AU, arbitrary unit; LPA, apolipoprotein(a); MW, molecular weight; ns, not significant; PCOLCE, procollagen C-endopeptidase enhancer 1.

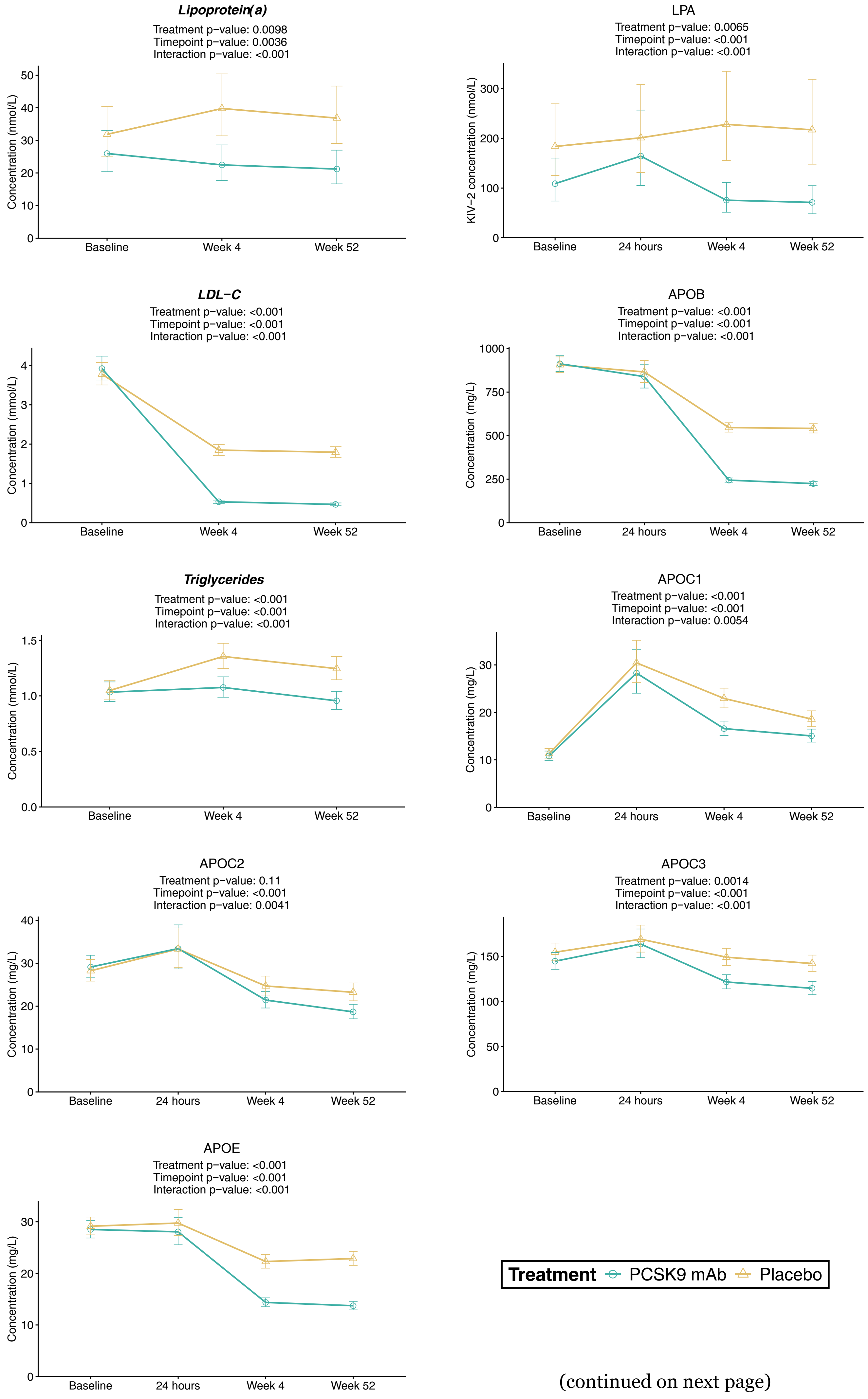

(continued on next page)

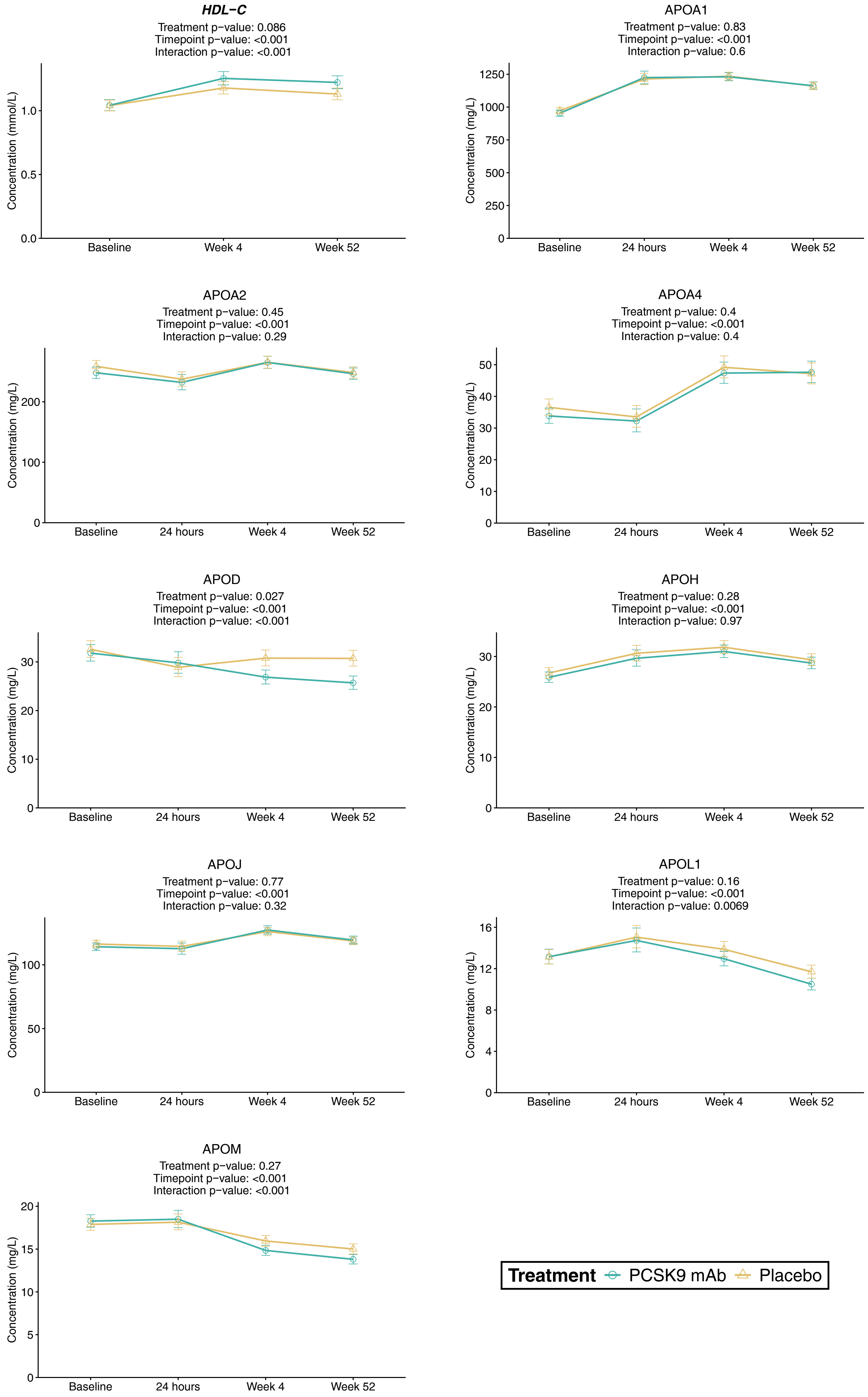

HDL−C
Treatment p−value: 0.086
Timepoint p−value: <0.001
Interaction p−value: <0.001
APOA1
Treatment p−value: 0.83
Timepoint p−value: <0.001
Interaction p−value: 0.6
APOA2
Treatment p−value: 0.45
Timepoint p−value: <0.001
Interaction p−value: 0.29
APOA4
Treatment p−value: 0.4
Timepoint p−value: <0.001
Interaction p−value: 0.4
APOD
Treatment p−value: 0.027
Timepoint p−value: <0.001
Interaction p−value: <0.001
APOH
Treatment p−value: 0.28
Timepoint p−value: <0.001
Interaction p−value: 0.97
APOJ
Treatment p−value: 0.77
Timepoint p−value: <0.001
Interaction p−value: 0.32
APOL1
Treatment p−value: 0.16
Timepoint p−value: <0.001
Interaction p−value: 0.0069
APOM
Treatment p−value: 0.27
Timepoint p−value: <0.001
Interaction p−value: <0.001
Concentration (mmol/L)
Concentration (mg/L)
Baseline
24 hours
Week 4
Week 52
Treatment
PCSK9 mAb
Placebo

Figure S6. Apolipoprotein levels over time in acute MI patients with corresponding clinical lipid measures: Lp(a), LDL-C, triglycerides, and HDL-C.
Values are marginal means with error bars indicating the 95% confidence interval. Green circles and lines indicate PCSK9 mAb, golden triangles and lines indicate placebo. Clinical lipids are displayed in bold italics. Number of patients per treatment and lipid variable (baseline, week 4, week 52): PCSK9 mAb (127, 127, 127), placebo (132, 132, 132). Number of patients per treatment and apolipoprotein variable (baseline, 24 hours, week 4, week 52): PCSK9 mAb (130, 38, 129, 129), placebo (133, 48, 131, 132). APO, apolipoprotein; HDL-C, high-density lipoprotein cholesterol; KIV-2, kringle IV type 2; LDL-C, low-density lipoprotein cholesterol; LPA, apolipoprotein(a); mAb, monoclonal antibody; MI, myocardial infarction; PCSK9, proprotein convertase subtilisin/kexin type 9.

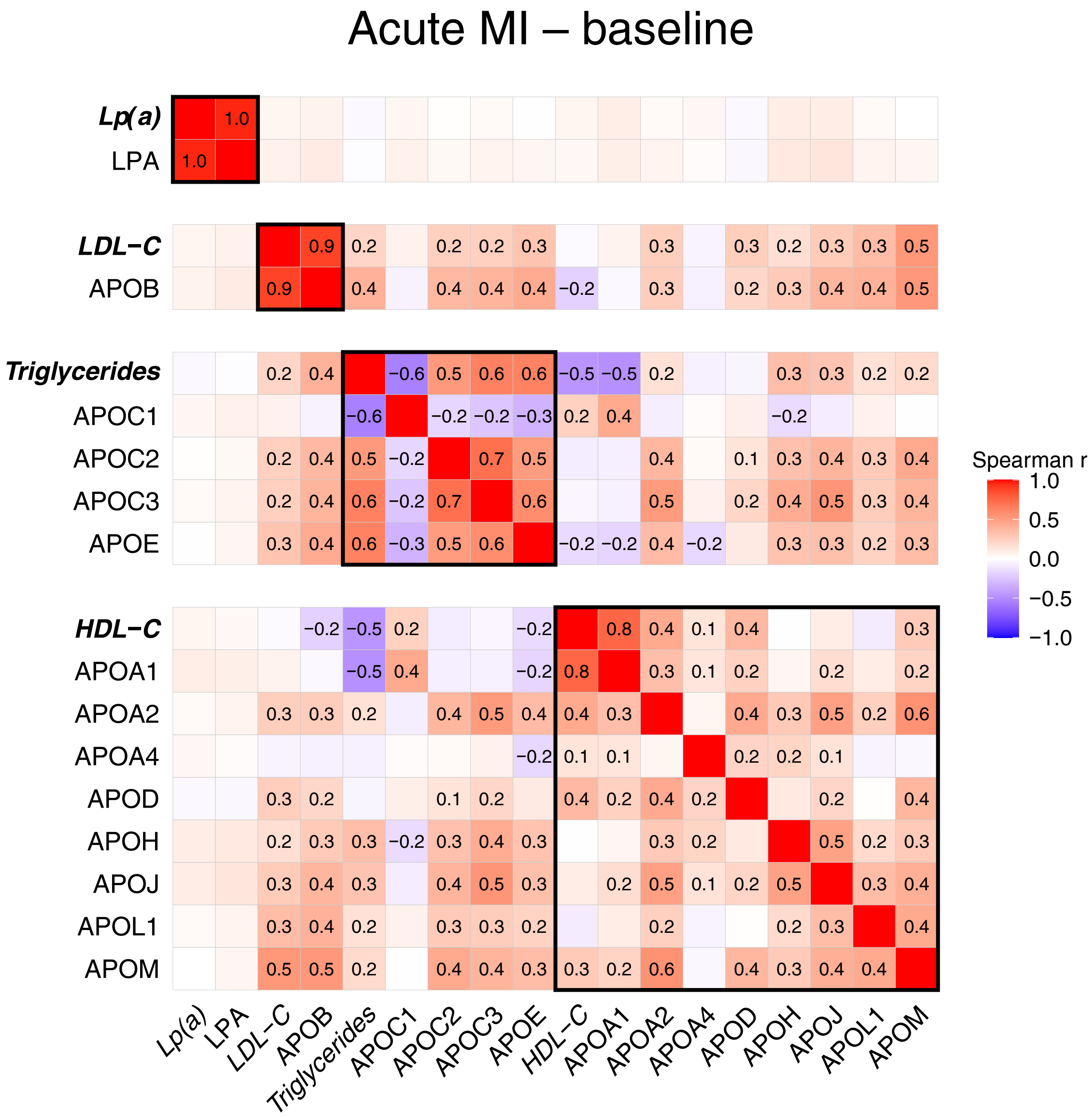

Figure S7. Baseline correlations between apolipoproteins and lipids in acute MI patients.

Spearman r values are indicated only for statistically significant correlations (adjusted P < 0.05). Clinical lipids are displayed in bold italics. P values were adjusted for multiple testing using the Benjamini-Hochberg method. Baseline plasma was unavailable for two acute MI patients from a total of 265. APO, apolipoprotein; HDL-C, high-density lipoprotein cholesterol; LDL-C, low-density lipoprotein cholesterol; LPA, apolipoprotein(a); Lp(a), lipoprotein(a); MI, myocardial infarction.

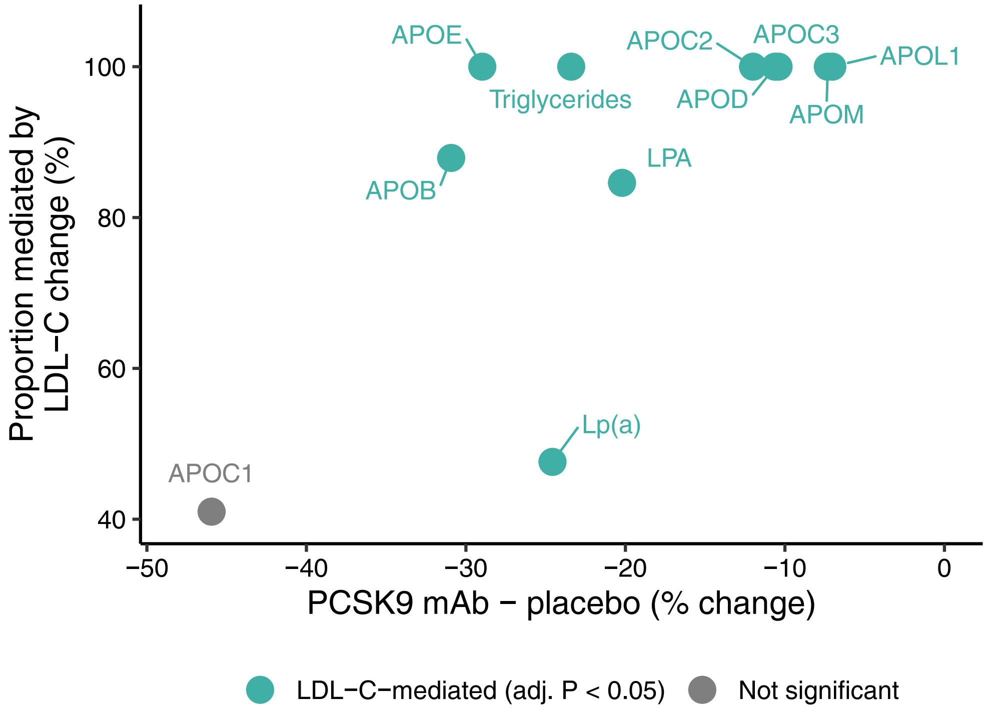

Figure S8. Scatter plot of mediation analysis including additional covariates. Compared with Figure 3C, the covariates age, sex, baseline statin status, and baseline LDL-C were included in both the mediator and outcome models. Parameters with significant mediation by LDL-C (adj. $P < 0.05$) are marked in green. All P values were adjusted for multiple testing using the Benjamini-Hochberg method. Adj., adjusted; APO, apolipoprotein; LDL-C, low-density lipoprotein cholesterol; LPA, apolipoprotein(a); Lp(a), lipoprotein(a); mAb, monoclonal antibody; PCSK9, proprotein convertase subtilisin/kexin type 9.

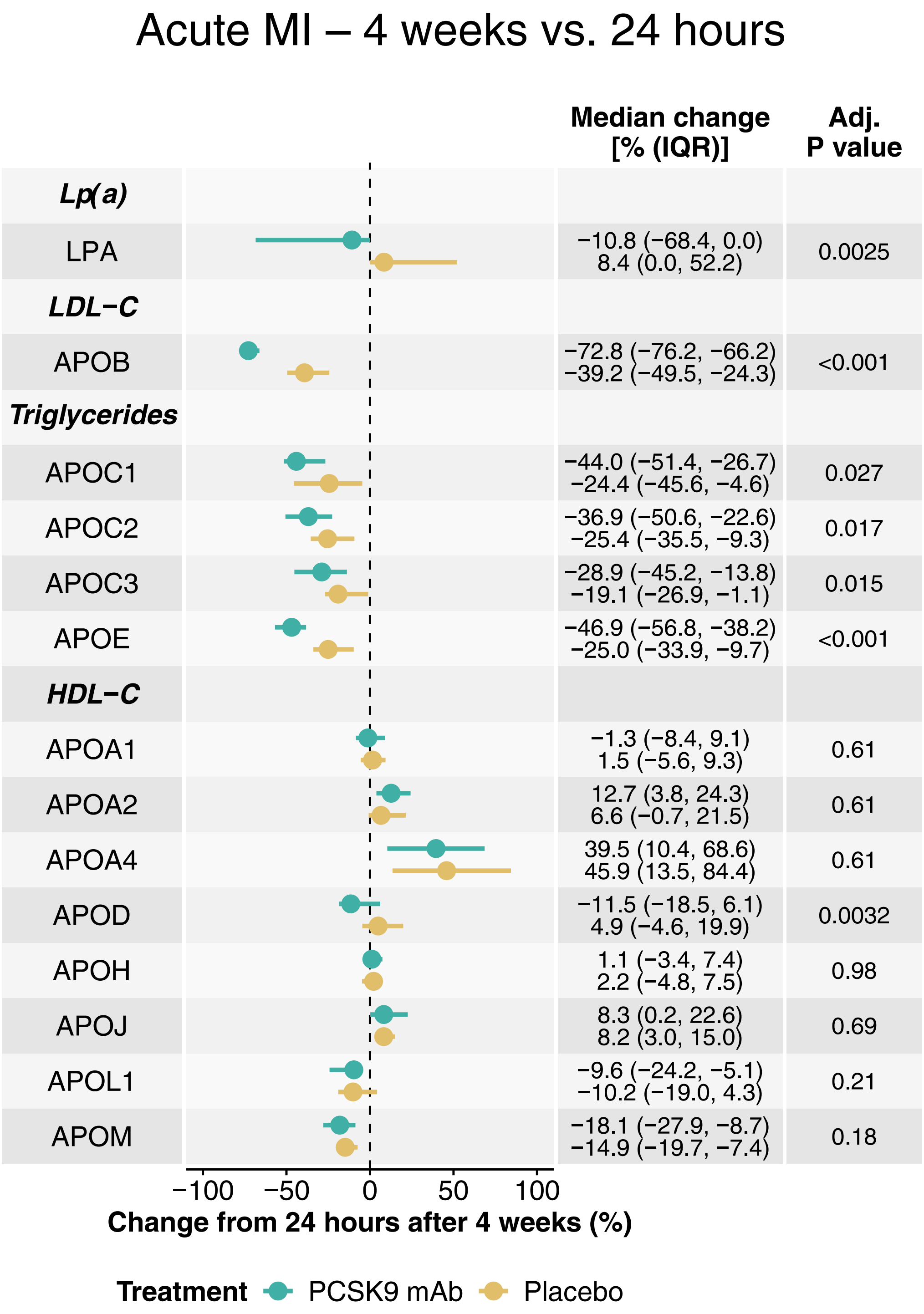

Figure S9. Apolipoprotein and lipid changes with PCSK9 mAb or placebo treatment.
Four-week changes with PCSK9 mAb (green) or placebo (gold) treatment in 85 acute MI patients of the PACMAN-AMI Bern subcohort with an additional 24-hour post-PCI blood draw. Clinical lipid measurements were unavailable at the 24-hour timepoint. Changes are median percent changes (25th, 75th percentiles). P values are from the Wilcoxon rank-sum test for between-treatment comparisons. Clinical lipids are displayed in bold italics. All P values were adjusted for multiple testing using the Benjamini-Hochberg method. Adj., adjusted; APO, apolipoprotein; HDL-C, high-density lipoprotein cholesterol; IQR, interquartile range; LDL-C, low-density lipoprotein cholesterol; LPA, apolipoprotein(a); Lp(a), lipoprotein(a); mAb, monoclonal antibody; MI, myocardial infarction; PCI, percutaneous coronary intervention; PACMAN-AMI, Effects of the PCSK9 Antibody Alirocumab on Coronary Atherosclerosis in Patients With Acute Myocardial Infarction; PCSK9, proprotein convertase subtilisin/kexin type 9.

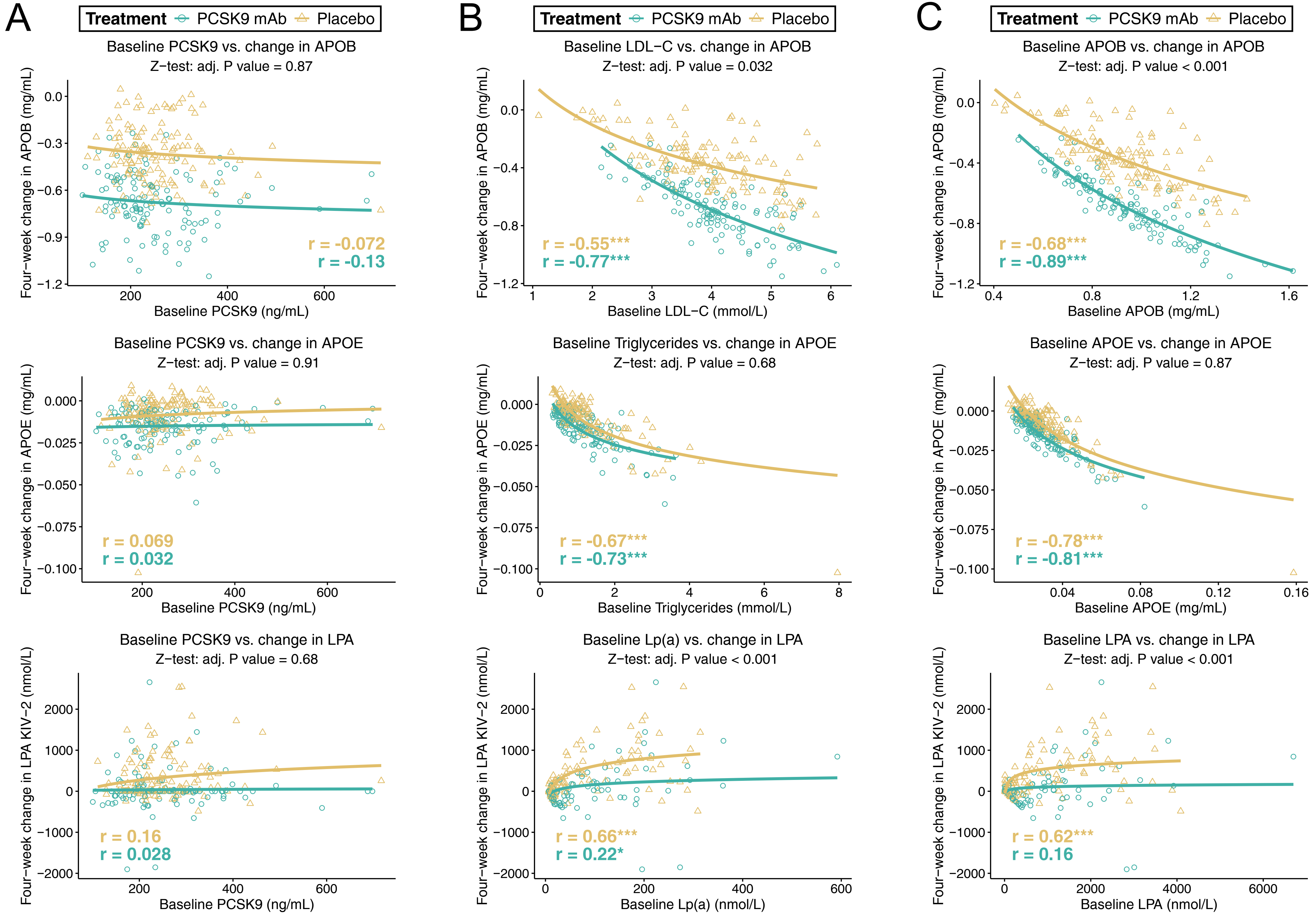

A
B
C
Treatment PCSK9 mAb Placebo
Baseline PCSK9 vs. change in APOB
Z−test: adj. P value = 0.87
r = -0.072
r = -0.13
Baseline LDL−C vs. change in APOB
Z−test: adj. P value = 0.032
r = -0.55***
r = -0.77***
Baseline APOB vs. change in APOB
Z−test: adj. P value < 0.001
r = -0.68***
r = -0.89***
Four−week change in APOB (mg/mL)
Baseline PCSK9 (ng/mL)
Baseline LDL−C (mmol/L)
Baseline APOB (mg/mL)
Baseline PCSK9 vs. change in APOE
Z−test: adj. P value = 0.91
r = 0.069
r = 0.032
Baseline Triglycerides vs. change in APOE
Z−test: adj. P value = 0.68
r = -0.67***
r = -0.73***
Baseline APOE vs. change in APOE
Z−test: adj. P value = 0.87
r = -0.78***
r = -0.81***
Four−week change in APOE (mg/mL)
Baseline PCSK9 (ng/mL)
Baseline Triglycerides (mmol/L)
Baseline APOE (mg/mL)
Baseline PCSK9 vs. change in LPA
Z−test: adj. P value = 0.68
r = 0.16
r = 0.028
Baseline Lp(a) vs. change in LPA
Z−test: adj. P value < 0.001
r = 0.66***
r = 0.22*
Baseline LPA vs. change in LPA
Z−test: adj. P value < 0.001
r = 0.62***
r = 0.16
Four−week change in LPA KIV−2 (nmol/L)
Baseline PCSK9 (ng/mL)
Baseline Lp(a) (nmol/L)
Baseline LPA (nmol/L)

Figure S10. Correlations between baseline protein or lipid levels and absolute treatment response in acute MI patients after four weeks.

A) Treatment response for APOB, APOE, and LPA depending on baseline PCSK9 levels.

B) Treatment response for APOB, APOE, and LPA depending on baseline LDL-C, triglyceride, and Lp(a) levels, respectively.

C) Treatment response for APOB, APOE, and LPA depending on baseline APOB, APOE, and LPA levels, respectively.

Treatment responses are absolute changes in 257 acute MI patients after four weeks of treatment. Green circles and lines indicate PCSK9 mAb, golden triangles and lines indicate placebo. Trendlines were fitted using linear regression on the logarithm of baseline concentrations. Significant Spearman r values are indicated by asterisks: ***, adj. P value < 0.001; *, adj. P value between 0.01 and 0.05. Z-tests were conducted to compare correlations. P values were adjusted for multiple testing using the Benjamini-Hochberg method. Adj., adjusted; APO, apolipoprotein; LDL-C, low-density lipoprotein cholesterol; LPA, apolipoprotein(a); Lp(a), lipoprotein(a); mAb, monoclonal antibody; MI, myocardial infarction; PCSK9, proprotein convertase subtilisin/kexin type 9.

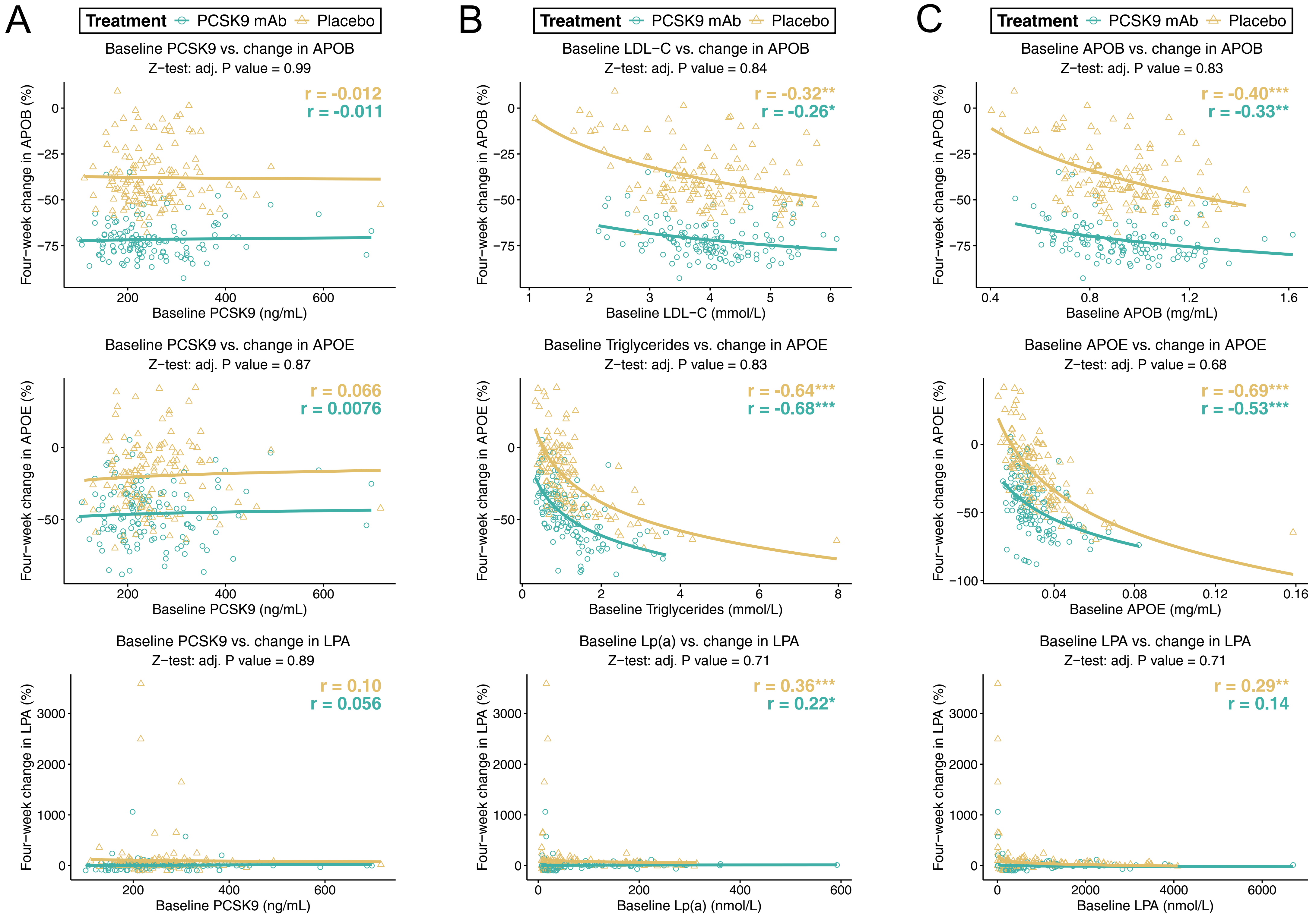
A
B
C
Treatment
PCSK9 mAb
Placebo
Baseline PCSK9 vs. change in APOB
Z−test: adj. P value = 0.99
r = -0.012
r = -0.011
Four−week change in APOB (%)
Baseline PCSK9 (ng/mL)
Baseline LDL−C vs. change in APOB
Z−test: adj. P value = 0.84
r = -0.32**
r = -0.26*
Baseline LDL−C (mmol/L)
Baseline APOB vs. change in APOB
Z−test: adj. P value = 0.83
r = -0.40***
r = -0.33**
Baseline APOB (mg/mL)
Baseline PCSK9 vs. change in APOE
Z−test: adj. P value = 0.87
r = 0.066
r = 0.0076
Four−week change in APOE (%)
Baseline Triglycerides vs. change in APOE
Z−test: adj. P value = 0.83
r = -0.64***
r = -0.68***
Baseline Triglycerides (mmol/L)
Baseline APOE vs. change in APOE
Z−test: adj. P value = 0.68
r = -0.69***
r = -0.53***
Baseline APOE (mg/mL)
Baseline PCSK9 vs. change in LPA
Z−test: adj. P value = 0.89
r = 0.10
r = 0.056
Four−week change in LPA (%)
Baseline Lp(a) vs. change in LPA
Z−test: adj. P value = 0.71
r = 0.36***
r = 0.22*
Baseline Lp(a) (nmol/L)
Baseline LPA vs. change in LPA
Z−test: adj. P value = 0.71
r = 0.29**
r = 0.14
Baseline LPA (nmol/L)

Figure S11. Correlations between baseline protein or lipid levels and relative treatment response in acute MI patients after four weeks.
A) Treatment response for APOB, APOE, and LPA depending on baseline PCSK9 levels.
B) Treatment response for APOB, APOE, and LPA depending on baseline LDL-C, triglyceride, and Lp(a) levels, respectively.
C) Treatment response for APOB, APOE, and LPA depending on baseline APOB, APOE, and LPA levels, respectively.
Treatment responses are relative percent changes in 257 acute MI patients after four weeks of treatment. Green circles and lines indicate PCSK9 mAb, golden triangles and lines indicate placebo. Trendlines were fitted using linear regression on the logarithm of baseline concentrations. Significant Spearman r values are indicated by asterisks: ***, adj. P value < 0.001; **, adj. P value between 0.001 and 0.01; *, adj. P value between 0.01 and 0.05. Z-tests were conducted to compare correlations. P values were adjusted for multiple testing using the Benjamini-Hochberg method. Adj., adjusted; APO, apolipoprotein; LDL-C, low-density lipoprotein cholesterol; LPA, apolipoprotein(a); Lp(a), lipoprotein(a); mAb, monoclonal antibody; MI, myocardial infarction; PCSK9, proprotein convertase subtilisin/kexin type 9.
